# Quantifying partisan news diets in Web and TV audiences

**DOI:** 10.1126/sciadv.abn0083

**Published:** 2022-07-13

**Authors:** Daniel Muise, Homa Hosseinmardi, Baird Howland, Markus Mobius, David Rothschild, Duncan J. Watts

**Affiliations:** ^1^ Department of Communication, Stanford University, Stanford, CA, USA.; ^2^ Department of Computer and Information Science, University of Pennsylvania, Philadelphia, PA, USA.; ^3^ Annenberg School for Communication, University of Pennsylvania, Philadelphia, PA, USA.; ^4^ Microsoft Research New England, Cambridge, MA, USA.; ^5^ Microsoft Research NYC, New York, NY, USA.; ^6^ Operations, Information, and Decisions Department, University of Pennsylvania, Philadelphia, PA, USA.

## Abstract

Partisan segregation within the news audience buffers many Americans from countervailing political views, posing a risk to democracy. Empirical studies of the online media ecosystem suggest that only a small minority of Americans, driven by a mix of demand and algorithms, are siloed according to their political ideology. However, such research omits the comparatively larger television audience and often ignores temporal dynamics underlying news consumption. By analyzing billions of browsing and viewing events between 2016 and 2019, with a novel framework for measuring partisan audiences, we first estimate that 17% of Americans are partisan-segregated through television versus roughly 4% online. Second, television news consumers are several times more likely to maintain their partisan news diets month-over-month. Third, TV viewers’ news diets are far more concentrated on preferred sources. Last, partisan news channels’ audiences are growing even as the TV news audience is shrinking. Our results suggest that television is the top driver of partisan audience segregation among Americans.

## Teaser

The American TV news audience is more partisan-segregated than the online news audience, cross-sectionally and longitudinally.

## INTRODUCTION

Echo chambers and filter bubbles have captured the imagination of academics and the public alike over the past decade. The rise of interest in these phenomena coincided largely with the adoption of social media, an era in which formerly passive information consumers could easily author, share, and reshare content of their own (*1*). Platforms, search tools, algorithms, and social networks have borne the task of curating an enormous and ever-increasing volume of information, including news content, that is now available online (*2*, *3*). As these curation systems rely in part on homophily (i.e., relative similarity of friends versus strangers), affinity (e.g., political partisanship), and demonstrated personal preference (e.g., previously viewed content), scholars have speculated that Americans’ news diets will, as a consequence, become less diverse, more segregated, and less likely to challenge existing opinions or to provide new perspectives (*1*). These general concerns are often expressed via the metaphor of the “echo chamber,” which refers to an online environment that allows for the exchange of content between similarly minded individuals, especially partisan content, at the exclusion of other perspectives (*4*, *5*). In recent years, scholars have also increasingly referred to “filter bubbles,” which are more specifically tied to the purported consequences of algorithmic ranking or recommendations (*5*–*7*). Broadly, however, both metaphors reflect similar underlying concerns about increasing online partisan audience segregation; thus, we refer to them interchangeably.

Partisan audience segregation and its subphenomena garner attention for good reason. Exposure to congenial news has been found to polarize already partisan individuals further to the left and right, both in terms of ideology and with respect to specific political issues (*8*, *9*). In contrast, it is thought that exposure to opposing views allows for reflection on one’s own viewpoints (*10*) and tempers political hostility toward political outgroups (*11*). News audiences that are segregated along partisan lines have comparatively less opportunity to become fully informed citizens and voters, a necessary component of a functioning democracy (*12*). Motivated by these concerns, numerous empirical investigations into the online media environment have been undertaken in recent years. Contrary to expectations, most of these studies have revealed limited evidence for echo chambers and filter bubbles (*13*–*21*): With a few exceptions (*20*, *22*), partisan audience segregation owed to platforms and algorithms has been found to act on a very small subset of the population (*13*–*21*), with presumably small downstream effects on the American democracy as a whole (*23*). This conclusion runs counter to the self-reported perceptions of Americans (*24*) but has birthed a reactionary line of scholarship, suggesting that expert and lay intuition about increasing online audience segregation is mistaken (*15*, *19*, *23*, *25*, *26*).

What is missing from this debate is a broader view of partisan audience segregation that includes the Internet but recognizes that the modal American experience of news cannot be adequately described or explained based on online behavior alone. Vastly, more news consumption occurs through ordinary television (TV)—not only cable and broadcast networks but also local news—than online (*27*, *28*). Moreover, TV news has long been subject to partisan bias. In particular, the advent of cable TV in the late 1980s (*29*) and its expansion through the 2000s (*30*) brought with it 24-hour partisan cable news channels, some of which have captured large audiences from middle-of-the-road broadcast networks like ABC, CBS, and NBC (*27*, *31*). Although cable news consumption is determined by viewer preferences, not background algorithmic decisions, the highly routinized programming style of prime-time cable news has some of the same properties as a partisan-biased ranking algorithm. Once viewers select into a partisan-biased news lineup (i.e., a news channel), simple inertia results in subsequent exposure to similarly biased content.

Here, we incorporate TV news into the literature on partisan audience segregation, providing direct comparisons against the online news audience and the U.S. population as a whole. To do so, we make use of two multi-year nationally representative panels, each comprising tens of thousands of American adults each month: a minute-level national TV tracking panel and a second-level laptop/desktop browser tracking panel. We analyze each panel independently and report the scale of partisan audience segregation via either platform as a percentage of the entire U.S. adult population, referred to herein simply as “Americans.” After corroborating that the TV news audience is much larger than the online audience (in terms of numbers and time spent viewing) and that online partisan segregation describes only a small fraction of Americans, we offer four key findings that together highlight the intensity of partisan segregation among TV viewers. First, we estimate that about 17% of Americans are partisan-segregated via TV—roughly four times as many as are partisan-segregated via online news consumption. Second, we address the persistence of partisan audience segregation over time for any given audience member, which has been mostly overlooked by prior studies. We find that TV news consumers are several times more likely to maintain their partisan news diets month-over-month. Third, by clustering all TV and Web audience members according to their news diets, we find that TV viewers’ news diets are far more concentrated on preferred sources, while even partisan online news audience members tend to consume from a variety of sources. Last, we examine the evolution of partisan news diets across 4 years and find that partisan cable news audiences are growing even as the whole TV news audience is shrinking. Overall, our results show that while only a minority of TV viewers are part of a partisan-segregated news audience, this minority is far larger and far more internally consistent than what has been found in the online media environment, algorithms or not.

Complementing our specific findings, we also outline a flexible but rigorous framework for identifying and measuring partisan audience sizes that can accommodate a variety of platforms, content sources, and definitions of partisan bias. We demonstrate this flexible framework by presenting our findings using lenient and strict approaches to estimating partisan audience segregation, along two dimensions. First, in determining whether an individual’s news diet is partisan biased in a given month, we estimate our findings under a lenient or strict criterion; respectively, 50 or 75% of the news diet must be composed of partisan-biased content, in terms of consumption time. Second, in determining which news content is considered partisan biased, we vary our criteria to include a larger or smaller (lenient or strict) array of programs or domains. Explanation of these parameters is available in Materials and Methods.

## RESULTS

**Fig. 1.**
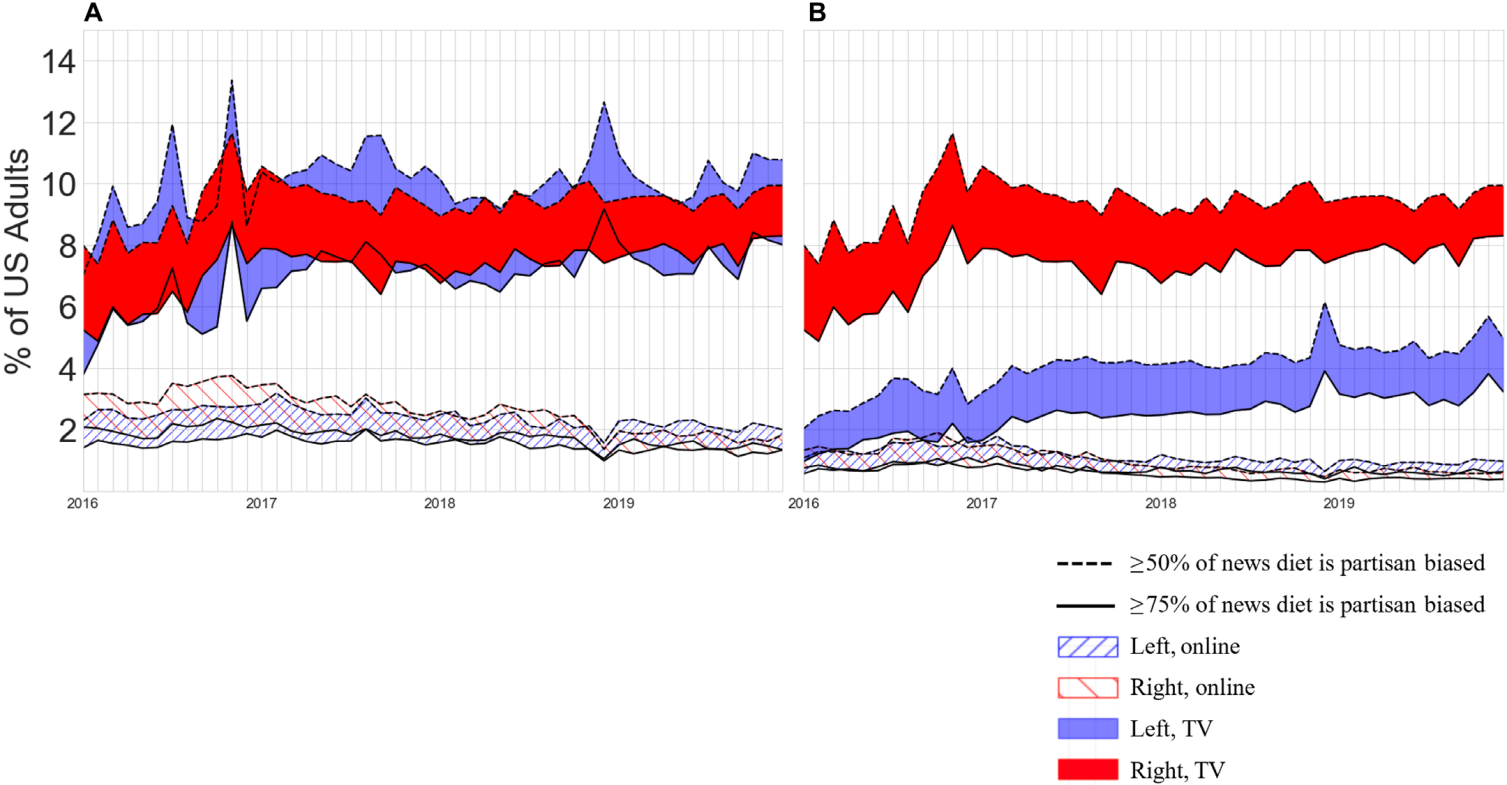
Partisan segregation in desktop and TV news audiences (2016–2019). The monthly percent of Americans experiencing partisan segregation via TV or Web news. Bounds (dotted and dashed lines) represent strict or lenient values of the percent of intra-individual news diets that must be partisan for partisan segregation (parameter 1). In (**A**) on the left, websites more partisan than TheGuardian.com (FoxNews.com) are counted as left (right). In stricter (**B**) on the right, partisan content bounds are Slate.com (Breitbart.com) on the left (right). CNN is counted as left-leaning in (A).

Our first main question concerns the relative scale of partisan audience segregation on TV and online. Figure 1 shows the percent of Americans partisan-segregated to the left or right on either platform using both lenient and strict approaches for news diet composition and news partisanship, respectively. In both panels, the vertical axis shows the percentage of Americans, and the horizontal axis shows each month from January 2016 and December 2019. Red coloration indicates the partisan right news audience, blue indicates the partisan left audience, solid coloration indicates the TV audience, and hatched fill indicates the online audience. The dashed line along the top of each band illustrates monthly partisan segregation estimates using a lenient definition of news diet composition, and the solid line along the bottom of each band indicates a strict definition. Thus, the fill between the two lines illustrates the complete range of possible values between the two parameterizations. Figure 1A, meanwhile, illustrates the lenient definition of news partisanship described above, i.e., MSNBC and CNN are counted as being on the left for TV, while online, anything more partisan than TheGuardian.com (FoxNews.com) is counted as left (right). Figure 1B, by contrast, illustrates the strict definition: Only MSNBC is counted as partisan left TV news, while anything more partisan than Slate.com (Breitbart.com) is counted as left (right).

Figure 1 corroborates existing research into the online media environment, showing for both lenient and strict definitions of partisan consumption that online partisan segregation is in the low single digits and shrinking since 2016 (*20*). In addition, Figure 1 shows that earlier findings that TV dominates online as a source of overall news (*28*) also apply to partisan news consumption. Specifically, Fig. 1A shows that partisan audience segregation affects between three and four times as many Americans via TV news as it does via online news, ranging as high as 23% of Americans at its peak in November 2016 (the month in which Donald Trump was elected) and ranging in the high teens for the following 3 years versus the partisan-segregated online audience, which ranges in single digits. Averaging across all 4 years, Fig. 1A shows that roughly 17% of Americans were partisan-segregated to the left or right via their TV news consumption, where partisan segregation in the TV news audience is fairly symmetrical between the left and right: 8.7% and 8.4% of Americans, respectively (it is more uneven for online news, but the absolute difference remains small). In contrast, excluding CNN from the partisan left, as we do in Fig. 1B, creates a substantial asymmetry in partisan TV consumption between right and left, essentially because the Fox News audience is roughly double that of MSNBC. Meanwhile, applying the strict definition of online partisan consumption creates no such asymmetry but further diminishes an already small population. In Fig. 1 (A and B), we note that, in addition to the peak of partisan audience segregation during the 2016 election, a second notable spike occurred in months leading into December 2018. This spike occurred asymmetrically for partisan left TV news and followed the 2018 “blue wave” midterm elections in which a record number of Democratic campaign advertisements were aired on TV (*32*). While our analysis is descriptive rather than causal, the placement of these two spikes suggests a clear connection between partisan content choices and events in the political arena.

Although partisan segregation is clearly higher for TV than for online, a question remains whether the average value of 17% (for our lenient definition) should be considered “large” or “small.” As with many such questions, the answer depends on the context. To illustrate, Prior (*33*) estimated that “10 to 15% of the voting-age population” were regular cable news consumers, implying that this was a small amount relative to the much larger population consuming network and other types of news. By contrast, the same number viewed in the context of the American electorate might seem much larger, especially considering that cable news consumption has a demonstrated causal effect on voter preference (*34*). For example, Guess (*20*) estimated that online partisan news consumers had a turnout rate slightly higher than the general turnout rate in the 2016 presidential election. Assuming a similar pattern for partisan TV news consumers, and assuming that they are eligible to vote at a similar rate as the general adult population (93%), then it follows that around 16% of votes in the 2020 presidential election were cast by partisan-segregated TV news consumers—a larger number than were cast in 24 states and the District of Columbia combined (*35*). This estimate would be even higher if we assume that partisan-segregated TV news consumers, being older and more educated than the general population, are somewhat more likely to vote (*36*). In summary, the scale of partisan segregation in the TV audience, 17%, could be viewed as small relative to popular fears of rampant polarization (*6*) but large relative to other politically consequential voting groups.

Moving beyond aggregate segregation, we next consider how partisan audience segregation varies across segments of the population. Following previous work on news consumption and platform choice (*2*, *22*, *28*, *37*), Table 1 shows a breakdown of our main results by age, race, and educational level, both for TV news (top) and online news (bottom). For each group and on either platform, we estimate the intragroup percentage that is partisan-segregated per month and average across the 4-year span of our data, where, again, we present results for both lenient and strict definitions of news diet composition and news partisanship. Considering age first, we follow a convention set by Allen *et al.* (*28*) to compare adults below 25 years old and adults 55 years old or older. As may be expected, we find that partisan segregation is much more apparent among older adults in the TV audience. However, Table 1 also shows that right-leaning partisan segregation in the online audience is also driven by older Americans: Adults 55 years old and older are five times as likely as young adults to be partisan-segregated, despite being much less likely to receive their news online (*38*). Similarly, under a strict definition of partisanship, we find that older adults are more likely than younger adults to be partisan-segregated to the left via online news. While our data cannot provide an explanation for this, we speculate that these findings are related to greater political interest and engagement among older Americans (*35*, *39*). Among young adults, a greater number are partisan-segregated via TV news versus online news, implying that TV news is a greater catalyst for partisan segregation regardless of a group’s likelihood for adopting new media.

**Table 1.** Partisan segregation size estimates by percent of demographic under multiple parameterizations and for various demographic cohorts. Intrademographic percentages are estimated according to the population size of the corresponding demographic in the United States. “Bias labeling strategy” determines which content is considered biased; “diet bias proportion” refers to viewers’ news diet composition. We source all population estimates from recent U.S. Census figures. For education levels, we restrict calculations to adults over 25, following the U.S. Census methodology (*56*). See table S6 for underlying count estimates.

**Partisan-segregated TV news audience sizes as percent of demographic**								
**Bias**	**Left**				**Right**			
Bias labeling strategy	Lenient		Strict			(Fox News)		
Diet bias proportion	0.5	0.75	0.5	0.75		0.5	0.75	
All adults	8.7	5.9	3.6	2.1		8.4	6.5	
White	7.3	4.9	3.3	2		10.3	7.9	
Non-white	12.7	8.8	4.3	2.4		2.9	2.1	
<25 years old	3.9	2.9	1.3	0.8		3.2	2.5	
55 years old+	11.9	7.8	5.8	3.3		14	10.9	
High-school diploma or less	4.9	3.1	1.8	1		6.5	4.9	
Some college	10.4	6.9	4.3	2.4		12.2	9.4	
College graduate	11.6	7.9	4.7	2.8		10.3	7.9	
Postgraduate	17.1	12.1	8	4.8		9.4	7.3	
**Partisan-segregated desktop news audience as percent of demographic**								
**Bias**	**Left**				**Right**			
Bias labeling strategy	Lenient		Strict		Lenient		Strict	
Diet bias proportion	0.5	0.75	0.5	0.75	0.5	0.75	0.5	0.75
All adults	1.9	1.1	0.9	0.5	2.3	1.4	0.9	0.5
White	1.8	1	0.9	0.5	2.7	1.7	1.1	0.6
Non-white	2.4	1.5	1.1	0.6	0.9	0.6	0.3	0.2
<25 years old	1.8	1.2	0.7	0.4	0.6	0.4	0.2	0.1
55 years old+	1.8	1	0.9	0.5	3.4	2.1	1.5	0.8
High-school diploma or less	0.7	0.4	0.3	0.2	1.2	0.8	0.5	0.3
Some college	2.7	1.6	1.3	0.7	3.7	2.4	1.5	0.8
College graduate	2.6	1.5	1.3	0.7	3.3	2	1.2	0.6
Postgraduate	3.1	1.6	1.5	0.8	3.1	1.7	1	0.5

Next, considering race, Table 1 shows that white Americans are far more likely to be partisan-segregated to the right on either platform, robust across lenient and strict approaches, than Americans who do not identify as white. The inverse is true on the left with less pronounced differences. The most extreme racial difference is in the right-leaning partisan-segregated audience; given our parameterization, this implies an association between race and loyal Fox News viewership. Last, considering education, Table 1 shows that the most partisan-segregated news consumers in our data are postgraduate degree holders on the left—a result that is robust to a range of parameterizations. Under our lenient approach to both parameters, as much as 17.1% of highly educated Americans are TV news consumers whose news diet is mainly left-leaning. Combined with the partisan right audience, more than a quarter of postgraduate Americans are partisan-segregated via TV news using our more lenient criteria. This result contrasts with self-reports suggesting that the education level bears no correlation to preference for watching cable news “often” (*40*) but comports with findings that at least college-educated Americans are more likely to engage in selective exposure to congenial news (*41*).

**Fig. 2.**
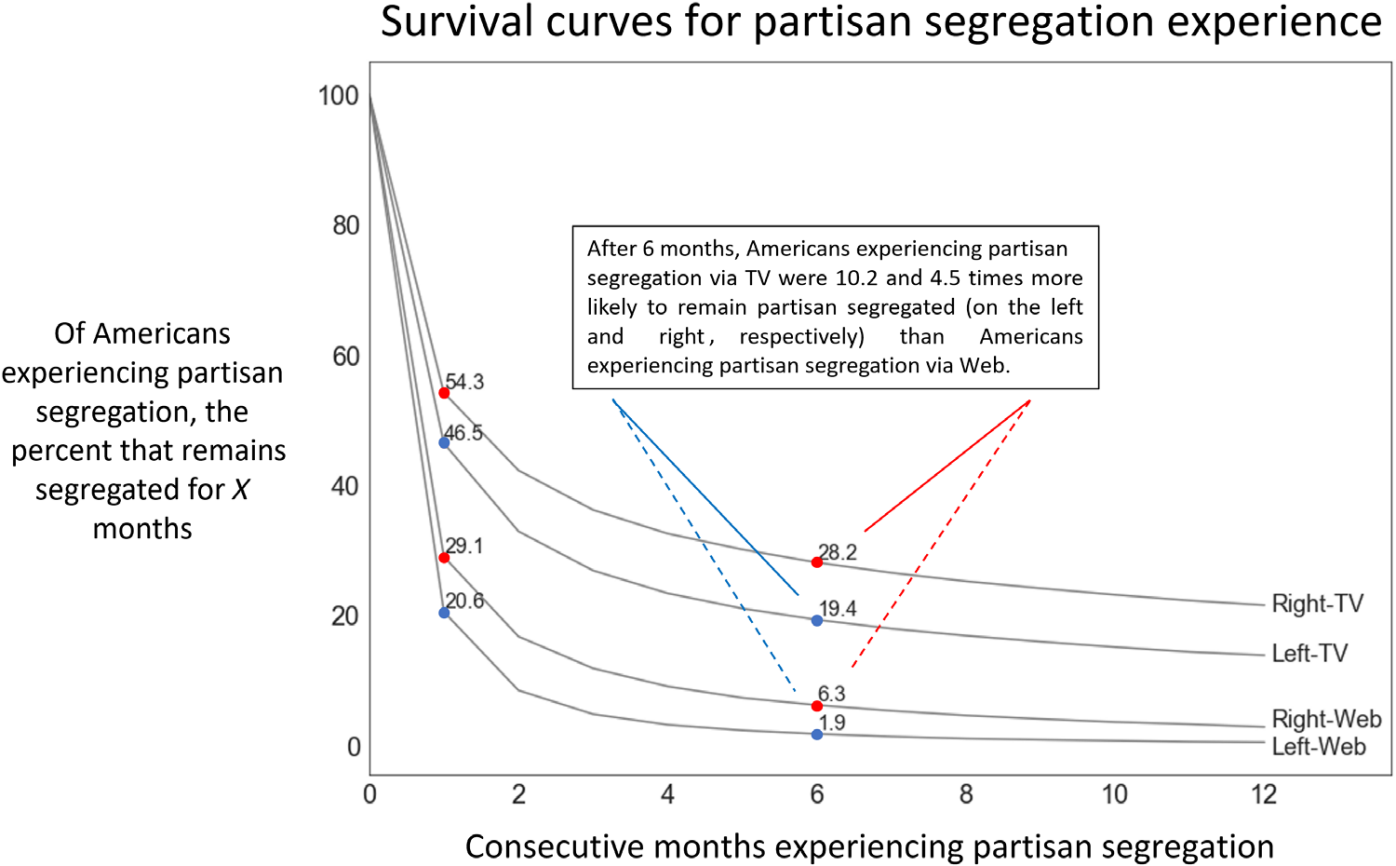
Survival curves for partisan segregation experience. Following lenient approaches to both minimum news diet and news partisanship used in Fig. 1A, we use the Kaplan-Meier estimator (*42*) to estimate month-to-month survival curves of partisan-segregated audience members for each of the four partisan-segregated audiences. These curves are conditional on a panelist having been identified as experiencing partisan segregation in at least 1 month. Table S6 in section S8 provides additional information and percentage values in terms of the American population.

Our second main question here regards the persistence of partisan segregation over time for the individuals that experience it. Although the metaphor of the echo chamber suggests that news audience members become trapped in a static environment, the reality of partisan audience segregation is repeated exposure to congenial news. The duration of this repetition is crucial to understanding the severity of partisan segregation: Partisan audience segregation estimates that are calculated only over a given cross-sectional time period or in aggregations over time as in Fig. 1 cannot capture intra-individual changes in news diets. To illustrate the importance of time in evaluating the severity of partisan segregation, imagine that 10% of a news audience is partisan-segregated in January, and 10% of that same news audience is partisan-segregated in February. On the basis of this finding, one might conclude that 10% of the population is partisan-segregated for the entire observation period, consistent with the conventional view of static echo chambers. However, it could equally be the case that the partisan-segregated audience members in February are entirely distinct from those in January, consistent with the less alarming view that high levels of partisan news consumption are transient and tend to be followed by exposure to other perspectives. To evaluate where, between these two extremes, partisan consumption “stickiness” sits, we examine the persistence of partisan news diets on either platform by applying right-censored survival analysis using the Kaplan-Meier estimator (*42*). We use this method to estimate the “survival” of partisan news diets as experienced by the individual news consumer, rather than by the whole audience in aggregate, given that any singular individual can alter their own news diet even as mass behavior remains stable and vice versa. Figure 2 shows survival analysis estimates for both TV and Web, where, in both cases, we have applied the lenient definition of news diet composition and also news partisanship. Four survival curves are shown in black with labels on the right indicating the news audience that each represents. On the horizontal axis, we show 12 months, where 0 indicates the first month that an individual qualifies as being partisan-segregated, and each subsequent number indicates consecutive months of maintaining the initial partisan news diet. On the vertical axis, we show the expected percentage of partisan-segregated Americans who maintain their news diets for as many consecutive months as is shown on the horizontal axis.

Overall, Fig. 2 shows that the intra-individual experience of partisan segregation is unexpectedly short-lived across all four audiences, relative to the static nature of partisan segregation implied by Fig. 1. The left-segregated online news audience is the most dynamic: When an online audience member first adopts a left-segregated news diet, there is only a 20.6% chance that they will maintain that news diet in the following month, a 1.9% chance after 6 months, and effectively 0% within a year. While right-segregated online news consumers are somewhat more likely to remain segregated after 1 month (29.1%), and more than three times as likely to remain after 6 months (6.3%), online partisan segregation—when it arises—is generally fleeting at the monthly level. Paralleling our earlier cross-sectional findings, Fig. 2 also shows that partisan segregation is considerably more persistent among TV audiences than online. After 6 months, TV audiences were 10 times and 4.5 times more likely to remain segregated than left and right online audiences, respectively (see sections S8 and S9 for more details). As with the cross-sectional findings, the survival rates of partisan segregation may be interpreted differently by different readers. On the one hand, the comparative stickiness of partisan-segregated TV news diets reinforces our earlier conclusion that at least some of the attention currently focused on online filter bubbles should be directed to TV. Whereas the online news environment offers thousands of choices, TV news viewers are made to choose from one of a handful of sources, which, once selected, act like a default choice (*43*) that must be actively altered. On the other hand, one might also take solace in the observation that even among TV viewers, somewhere between 70% (for right partisan viewers) and 80% (for left partisan viewers) do switch within 6 months. To the extent that long-lasting echo chambers do exist, therefore, they include only about 4% (0.25 × 17 = 4.25) of the population.

Returning to cross-sectional analysis, we now dig more deeply into the composition of American news diets by identifying archetypes of news consumption that allows us to (i) examine the internal consistency of news diets among all four partisan audiences and (ii) better understand the news diets of Americans who do not qualify as partisan-segregated. To construct archetypes, we first bucket the ranked spectrum of left-to-right news websites into five categories (furthest left to furthest right) according to our lenient and strict thresholds for news partisanship and “portals” such as MSN.com. These categories of websites are treated as six independent dimensions for each panelist, measured by minutes spent viewing each category of websites in an average month. Next, we apply the Louvain unsupervised clustering algorithm to find clusters of browser panelists with similar online news diets (*44*). This community detection technique allows us to group audience members according to their intra-individual allocation of time across news sources, providing a comprehensive view of archetypal news consumption behaviors in the entire audience (see section S10 for more details). Last, we define the archetype for a cluster c as the average news consumption distribution of all individuals in c. The same algorithm is applied to identify archetypes of TV news consumption, starting with seven categories of news programming—MSNBC, CNN, Fox News/Fox Business News, hard news on broadcast stations, soft news on broadcast stations, Spanish language news programming, and remaining cable news programming aggregated together. More details are available in sections S2 and S4.

**Fig. 3.**
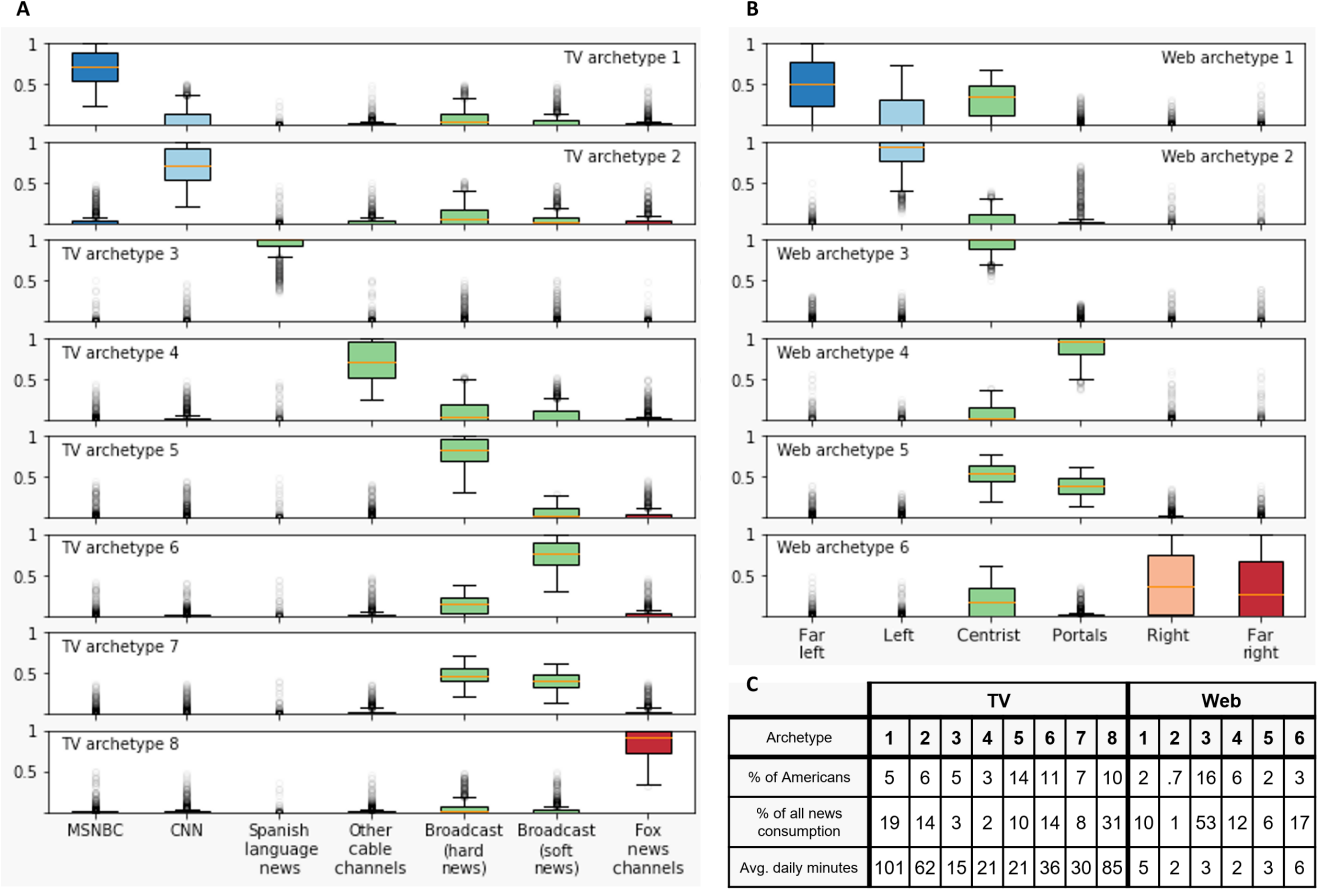
Archetypal news consumption behavior in the American audience. Panels (**A**) and (**B**) respectively show archetypal news consumption via television and online, and their scale is shown in Panel (**C**). By clustering panelists in terms of their distribution of consumption over the dimensions of content and identifying the centroid news consumption pattern in each cluster, we identify six archetypal online news diets and eight archetypal TV news diets. The archetypes each represent the respective average distribution of content consumed by all people in the corresponding cluster. To qualify as a member of any archetype, panelists must consume a minimum amount of news per month (30 min/month and 2 min/month on TV and Web, respectively, parameter 3); the first row of (C) does not add up to 100 due to the percentage of Americans who do not meet this criterion.

Figure 3 shows the results of our archetyping algorithm for TV news (left) and online news (right). In both cases, each row of box plots represents an archetype, and each column represents a news source, as indicated by coloration and by labels along the bottom of the figure. Every news audience member is assigned to the archetype that best represents their own news diet in terms of the intra-individual proportion of time they spend consuming news from each source category. Each individual box plot illustrates the range of intra-individual news diet proportions that cluster members allocate to a particular source category. On both media platforms, the predominant pattern of news consumption is centrist and mainstream. In the online audience, centrist and mainstream news comes mainly from moderate websites (e.g., USAToday.com), as in online archetypes 3 and 5, and portal websites (e.g., Google News), as in online archetypes 4 and 5. In the TV audience, it mainly comes from hard news broadcast programs (e.g., NBC Nightly News), as in TV archetypes 5 and 7, and soft news broadcast programs (e.g., America This Morning), as in TV archetypes 6 and 7.

Figure 3 reveals that TV audience members adhere more closely to their most preferred source category than online audience members do in Fig. 3B, particularly among partisan TV news viewers. For example, Fig. 3A shows that most Americans who consume mostly MSNBC rarely consume news from any other source besides CNN (archetype 1), while most Americans who consume mostly Fox News do so at the expense of all other sources (archetype 8). The only archetype with sharper source exclusivity than archetype 8 is TV archetype 3, which shows that, virtually, all viewers of Spanish language do not consume news from any other category. The source exclusivity seen in the TV audience is in clear contrast with online news diets shown in Fig. 3B. For example, the most left-leaning group of online audience members (archetype 1) still gets a sizable amount of news from centrist sources as does the most right-leaning group (archetype 6), albeit to a lesser extent than archetype 1. Together, Fig. 3 reinforces and complements the findings of Figs. 1 and 2: In addition to being larger and more persistent, partisan TV news audiences demonstrate more exclusive preferences for partisan content than do their online counterparts, which tend to include some centrist sources in their news diet. In addition to corroborating the relative scale of partisan segregation across platforms (i.e., Fig. 1), this finding also illuminates a possible partial explanation for rapid turnover in online partisan news diets identified in Fig. 2. The large array of sources available to online news consumers allows for news diets to fluctuate back and forth over a threshold of partisan news exclusivity, whereas the relative paucity of choices available on TV encourages more concentrated consumption.

In addition to shedding light on the concentration of partisan consumption across archetypes, Fig. 3C shows that Americans who belong to the most partisan TV archetypes are also the most voracious consumers of news. For example, audience members assigned to (left-leaning) archetype 1 watch nearly 2 hours of news on an average day, while those in (centrist) archetype 5 watch an average of just 21 min/day. Overall, Americans belonging to TV archetypes 1, 2, and 8 comprise 21% of the population but account for three times as much (64%) of all news minutes consumed. The online news audience shows an even larger proportional effect of partisanship on consumption: 6% percent of Americans belong to the most partisan Web news archetypes (1, 2, and 6) but account for five times as much (28%) of all online news minutes consumed. We note, however, that the total fraction of consumption attributable to highly partisan online archetypes is still less than half of the corresponding fraction via TV. Viewed differently, online archetype 3, which is almost exclusively centrist, comprises 53% of consumption time, whereas TV archetypes 5 to 7 (broadcast news) comprises only 32%. Considering that TV news consumption is much larger in absolute terms than online news consumption in terms of viewing hours (*28*) and also that the partisan TV news audience alone consumes more minutes of news than does the entire online news audience, it follows that highly concentrated partisan news consumption is a much bigger phenomenon on TV than online (see section S10 for more details).

**Fig. 4.**
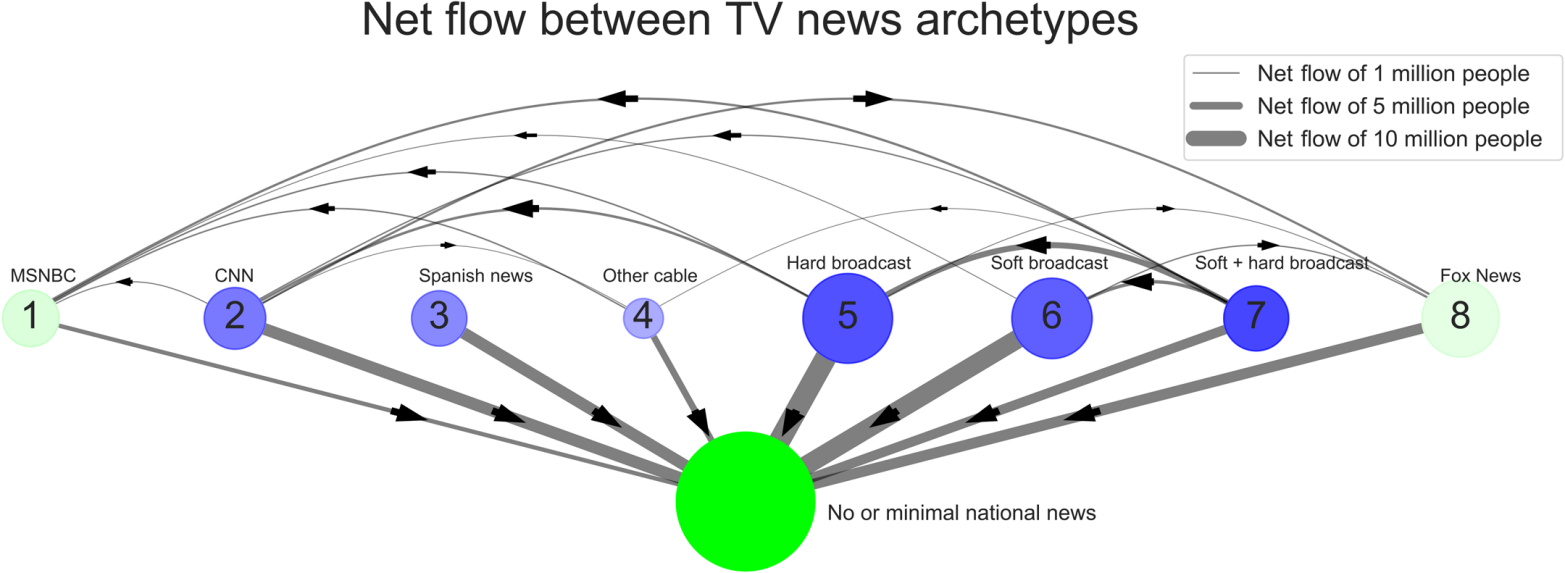
The “net flow” of people between and out of the eight television news archetypes. Television news consumption archetypes, as seen in Fig. 3A, are each labeled according to the category of news most prominently consumed within the archetype, along with a large ninth group of people who are exposed to less than 30 min of news in a month, calculated independently in each month over the 4 years of analysis. Net flow represents the direction and magnitude of turnover between a pair of archetypes. Specifically, if we let $A_{i}^{k}$ be the set of people in archetype *i* during month *k*, the net flow between archetypes *i* and *j* is defined as the absolute value of the expression $|A_{i}^{k}-A_{j}^{k+1}|-|A_{j}^{k}-A_{i}^{k+1}|$ summed over all pairs of months (*k,k + 1*). The direction of the net flow, signified by the arrows, points toward group *j* if net flow—before absolute value—is positive and toward group *i* if it is negative. We do not show net flows of less than 1 million people. Node diameter corresponds to the size of the population of the archetypal cluster averaged over all months. Green signifies that an archetype has experienced net inflow, while blue signifies net outflow, with a levels corresponding to the scale of net in(out) flow.

Having established that partisan segregation is more prevalent, more concentrated, and more persistent on TV than it is online, our fourth and final question concerns the evolution of partisan audiences exclusively on TV between 2016 and 2019. To address this question, we disaggregate each panelist’s TV news diet into 48 monthly snapshots and then, for each month, assign them to the archetype that best matches their news diet for that month. Each panelist can now be treated as a time series of sequential monthly archetype assignments, which may or may not change over the time period depending on the panelist. Figure 4 shows the net flow of Americans between archetypes from January 2016 to December 2019. Each blue or green node represents a TV archetype, labeled numerically according to Fig. 3 and descriptively according to the most common source category in the archetype. Nodes shaded in blue indicate archetypes that have shed audience members over time, and nodes shaded in green indicate archetypes that have gained audience members over time. Node opacity indicates the scale of new flow, and node diameter indicates the scale of the cluster averaged across all 48 months. Net flows between clusters are shown as edges, with thickness indicating the flow size and arrows indicating the directionality.

In light of our earlier findings regarding the dominance of TV over online news, a notable message of Fig. 4 is that people from every group are turning away from national TV news in substantial numbers. The exodus is proportionally greater from centrist archetypes such as hard broadcast and least from the two most partisan archetypes, archetypes 1 and 8. Within the remaining TV news audience, there has been movement from broadcast news to cable news, ultimately trending toward MSNBC and Fox News Channel. We also see that fewer Americans are consuming news diets composed of both soft news and hard news from broadcast stations instead opting for one or the other. Together, Fig. 4 reveals a counterintuitive finding: Although the overall TV news audience is shrinking, the partisan TV news audience has grown in absolute terms. This helps explain how the scale of the partisan-segregated TV audience shown in Fig. 1 remained stable over time despite viewers shifting away from the platform. Broadly, the TV news audience is undergoing a distillation process, whereby remaining viewers are increasingly partisan, and the partisan proportion of TV news consumers is on the rise. More details, and an analogous diagram of the net flow between online news archetypes, are available in section S10.

## DISCUSSION

Here, we have expanded the scope of partisan audience segregation by moving beyond discussions of exclusively online echo chambers and filter bubbles to incorporate the comparatively larger TV news audience. We present four main findings. First, we find that a far larger share of Americans is partisan-segregated via their TV news consumption (17.1%) than is partisan-segregated via their online news consumption (4.2%). Second, we find that partisan news diets are generally temporary on either platform but are more persistent on TV than online: While partisan TV news diets have a roughly one in four chance of lasting 6 months, partisan online news diets have a roughly 1 in 20 chance of lasting that long. Third, we find that news diets among the TV news audience are much more heavily concentrated on preferred sources, especially partisan sources, compared to news diets in the online audience. Fourth, we find that the partisan TV news audience is growing in absolute terms even as the entire TV news audience is shrinking.

Although our work sheds new light on the prevalence, concentration, and persistence of partisan segregation across online and TV news, it also exhibits important limitations. First, our data, while extensive, are incomplete in potentially important ways. For example, it does not account for online news encountered on smartphones or outside the Web browser, including sources such as radio and direct news consumption on social media. According to previous work (*28*), smartphones and social media news consumption behavior is similar to news consumption on desktop and hence should not be expected to change our overall conclusions; however, greater clarity on cross-platform media repertoires would illuminate media consumption more completely. We also do not incorporate America’s large array of local news outlets and audiences into our analysis. Growing nationalization and partisan influence in local news programming suggests that local news may have more influence on partisan segregation as it evolves (*8*, *45*) and hence is a natural direction for future study. YouTube is also an important growing source for partisan-skewed news (*46*, *47*), meriting further investigation into the scale of political information dissemination occurring on the platform. Second, our methods for identifying the partisan bias of news rely on domain-level source attribution. Contemporary research suggests that domain-level aggregation masks some user-level preference for sharing articles that are closer to each users’ own bias relative to the average bias of the article’s source. This effect is slight, even in sharing behavior, which is presumably more skewed than the consumption behavior we measure here (*48*). To the extent that domain aggregation affects our measurement of partisan segregation, we assume that it similarly affects our measurement of partisan segregation via TV news (where we mainly aggregate by channel rather than program). Working in the opposite direction, not all content in partisan domains and channels is partisan: While some audience members of partisan sources may seek out the like-minded material in a given publication, others may actually be exposed more heavily to centrist or opposing views. The implication is that with cross-pressure, we may slightly underestimate and overestimate partisan segregation on both platforms simultaneously, but our substantive findings are otherwise unaffected. With sufficient computational resources and advanced content analysis techniques of transcript data, future researchers may be able to describe units of content with greater granularity without relying on audience-level bias estimates, assuming that intrasource partisan bias is meaningfully varied.

Further, our descriptive results raise questions about explanatory mechanisms that we are unable to answer. For example, the survival analysis conducted in Fig. 2 demonstrates that partisan segregation is fairly short-lived relative to the duration of our sample, lasting only a few months for most audience members, and relatively shorter for Web news viewers. Unfortunately, our data are not well suited to answering why this is the case, but future work may seek to disambiguate between several possible explanations. For example, the comparative stickiness of TV consumption may derive in part from the passive nature of TV viewing, which contrasts with the more active nature of online consumption. Alternatively, it may arise simply from the relatively small selection of sources available via TV vis-a-vis online, as we have already speculated. Yet, another alternative might be that traffic to news websites is driven in part by clicking on links to articles from social media or news aggregators, while TV is still mostly navigated by selecting a channel and seeing what is on (although with modern menus, even news programs are increasingly unbundled). Yet, another might be that there is greater brand affinity for TV channels than Web publishers. Disambiguating between these individually plausible explanations would be interesting but would require a mix of experimental and survey research designs beyond the scope of panel data. Similarly beyond scope are the mechanisms driving the exodus from TV news in Fig. 4. For example, does it represent “cord cutting,” in which the same content is being viewed via Web streaming services and hence is no longer being registered as “TV,” or does it represent genuine disengagement with news? Last, our data and hence our conclusions are limited entirely to the United States, but similar questions could be asked of many other countries, potentially with different results. For example, as recently as 2014, TV news was found to be consistently very popular across 56 countries (*27*), while online polarization abroad seems to share both similarities and differences with the American case (*49*).

These unresolved issues notwithstanding, we close by noting that any resulting inferential errors would have to be very large to alter our main conclusions regarding the relative state of partisan-segregated news consumption online and on TV. With respect to online, partisan-segregated news consumption affects at most a few percent of Americans (Fig. 1), only a few percent of those people remain partisan-segregated for more than a few months (Fig. 2), and even those few percent consume a nonnegligible fraction of nonpartisan news. Although highly concentrated consumption of highly partisan material can be a cause for concern even if it affects only a small number of people—if, say, it facilitates extremist or violent behavior—longstanding concerns about the supposed ubiquity of online filter bubbles are not supported. With respect to TV, the picture is more complicated. On the one hand, our results make clear that partisan-segregated consumption is far more prevalent on TV than it is online, affecting as much as 17% of the population (Fig. 1). It is also considerably stickier and more concentrated: After 6 months, the fraction who remain partisan-segregated is several times larger than it is online (Fig. 2), and the inhabitants of the most partisan archetypes consume partisan content almost exclusively (Fig. 3). On the other hand, whether even these much larger numbers should be considered large depends very much on what they are being compared with. If one were under the impression that the entire country was living in echo chambers, for example, 17% might sound reassuringly small. Likewise, if one had assumed that an echo chamber, once inhabited, was a permanent state of affairs, then it might be reassuring to learn that three quarters of inhabitants had left after 6 months. However, viewed from another perspective—say the percentage of voters needed to sway an election—17% may seem like a very large number indeed. Between these differing interpretations, our own is that partisan segregation in TV audiences—whether it is large enough to be considered alarming—is large enough to justify TV news receiving at least the same level of scrutiny as its online counterpart.

## MATERIALS AND METHODS

Our main source of data comes from the Nielsen Company. Nielsen maintains large, representative panels of American households, the members of which agree to have their media habits tracked in exchange for payment. This study makes use of two such panels, differentiated by the type of media being tracked: a minute-level national TV panel (around 85,000 Americans in an average month) and a second-level laptop/desktop Web browsing panel (around 60,000 Americans in an average month), where users remain in-panel for several consecutive months before rotating out. The two datasets comprise over 3 billion unique viewing and browsing events. Smartphone news consumption and online streaming news are not included in our analysis but follow a similar pattern as news consumption on the desktop browser (*28*) and have a similar bias pattern. Following convention, we proxy meaningful social media news encounters by capturing news URLs (Uniform Resource Locators) outlinked from social media (*28*). As our focus is on national news, local TV news programming is not included in our analysis, but syndicated content on network affiliate stations is (e.g., Good Morning America shown outside of ABC’s main national channel).

To assign partisan bias labels to news websites, we draw on prior work that assigned ordinal left-to-right rankings to Web domains based on audience sharing behavior on Twitter (*18*). Recognizing the theoretical limitations of audience-based bias rankings, we validate these scores against two other sources that used different methodologies (*13*, *16*), finding a high correspondence between them (see section S3 for details). To assign partisan bias labels to TV news programs, we first group content into categories corresponding to the major channels that air news. MSNBC, Fox News, and CNN are by far the largest cable news channels, each with largely consistent bias in their programming, relative to one another: left, right, and center/left (*34*). News content on the “big three” former broadcast stations (ABC, CBS, and NBC) are grouped together based on format similarity (*50*). Given their common reliance on mainstream news agendas, they are jointly considered centrist relative to the three major cable news channels (*51*, *52*). Later, here, we separate former broadcast stations’ news programs into hard or soft broadcast news, i.e., airing primarily or partially political content, respectively. News programming from smaller stations with comparatively negligible news program viewership (e.g., Al-Jazeera America, AON, BET, and HLN) forms a “long tail” and is grouped together in a miscellaneous category rather than individually sorted into left and right buckets (see sections S3 to S5 for details).

To measure partisan audience segregation, we first estimate the scale of left-leaning or right-leaning partisan segregation on either platform as a percent of Americans. Here, we emphasize that there does not exist any objective, or even universally agreed upon, definition of what constitutes “left-leaning” or “right-leaning” content or what it means to be a “consumer” of this content. Any operational definition that one chooses is therefore necessarily arbitrary to some degree, where different definitions may appear more or less defensible to different observers under different objectives. Moreover, different definitions could produce different quantitative, or even qualitative, conclusions regarding the scale and persistence of echo chambers. As we will show, for example, whether one counts CNN as “left leaning” or not can substantially change one’s conclusion about the fraction of Americans whose TV consumption is segregated along partisan lines. Similarly, we will show that different assumptions regarding the fraction of time one must spend consuming homogeneously partisan content can change one’s quantitative conclusions regarding the scale of partisan segregation, but that qualitative conclusions are robust to these changes. Recognizing the potential for analytical ambiguity and its potential consequences for our substantive conclusions, we adopt a deliberately flexible framework for reporting our results as a function of two key variables that encode different assumptions of what partisan audience segregation means.

1) Minimum news diet composition: How much of an individual’s news diet, measured as a percent of one’s total time spent consuming news online or on TV each month, must be clearly partisan for that individual to be considered “partisan-segregated”? Here, we define two thresholds, “lenient” and “strict,” recognizing that other definitions could also be made. According to our lenient threshold, a news diet is considered partisan left or partisan right if a simple majority of news content is biased left or right, respectively, whereas our strict threshold requires that at least 75% of a whole news diet must be partisan. Reflecting our general approach to analysis, in Fig. 1, we apply both strict and lenient thresholds and present both sets of results to allow for comparison.

2) Determination of news partisanship: Which news content can be counted as partisan? For TV, we answer this question at the level of channels. In all cases, we treat programming from Fox News (and Fox Business) as partisan right and MSNBC partisan left (*34*); however, we allow CNN to be classified alternatively as partisan left or centrist (i.e., neither right nor left), reflecting its classification in prior research (*17*, *53*–*55*). For online news domains, the answer depends on a pair of threshold websites on the ordinal bias spectrum, one on the left and one on the right, such that all domains beyond these thresholds are counted as partisan biased. Once again, we do not suggest that any precise choice of threshold is objectively correct; thus, we again propose lenient and strict definitions of “left” and “right” to indicate a range of plausible conclusions. A lenient approach would include moderately partisan sources as biased, while a strict approach only assigns partisan bias to sources that are more clearly left-leaning or right-leaning. Following our general approach, we first report our results using both strict and lenient threshold choices, noting that, as with our definitions for TV, both choices reflect prevailing opinions regarding the partisanship of popular sources.

In addition to minimum news consumption and partisanship, we fix the values of two other variables to which our results are less sensitive: minimal news consumption, defined as the number of minutes per month for an individual to be included in our analysis, and time scale, defined as the interval over which we measure each individual’s partisan consumption. Choosing a minimum threshold for news consumption is necessary to avoid classifying panelists as belonging to an echo chamber when they consume only a tiny amount of total news (e.g., 20 s) each month. Meanwhile, the particular choice of time period over which to compute the fraction of partisan versus total news necessarily strikes a balance between too short (in which case conclusions may be biased by random variations in intraday or interday consumption patterns) and too long (in which case movement into or out of partisan news diets is obscured). We fix the minimum monthly news consumption at 30 and 2 min for TV and Web news consumption, respectively, and set the time scale at 1 month, emphasizing that our results are robust with respect to these choices (see sections S7 and S9).

## Supplementary Materials

### This PDF file includes:

Supplementary Text Figs. S1 to S6 Tables S1 to S6 References
