# Supplementary material for: Quantifying partisan news diets in Web and TV audiences
Source: Sci Adv. 2022 Jul 13;8(28):eabn0083. doi: 10.1126/sciadv.abn0083 (PMC9278856; doi:10.1126/sciadv.abn0083)
Supplement: Supplementary file 1 — Supplementary Text Figs. S1 to S6 Tables S1 to S6 References [file sciadv.abn0083_sm.pdf]

Science Advances
AAAS

Supplementary Materials for

Quantifying partisan news diets in Web and TV audiences

Daniel Muise *et al.*

Corresponding author: Daniel Muise, muise.dan@gmail.com

Sci. Adv. **8**, eabn0083 (2022)
DOI: 10.1126/sciadv.abn0083

This PDF file includes:

Supplementary Text
Figs. S1 to S6
Tables S1 to S6
References

1. Data source & details

Our data comes from the Nielsen Company. The Nielsen Company maintains large panels of American households which agree to have their media habits tracked in exchange for payment. This study makes use of two such panels, differentiated by the type of media being tracked: national television and laptop/desktop web-browsing. We make use of four continuous years of panel data. Both panels have rotating membership. The maximum amount of time that any individual remained in either panel was 24 months. In total, there were roughly 350,000 unique participants in the television panel, and roughly 500,000 unique participants in the browser-tracking panel. In a given month, the panel had an average of roughly 100,000 and 60,000 active participants for television and browsing respectively. Altogether, our data included over three billion unique events of viewing or browsing activity. Throughout the main text and these Supplementary Materials, we analyze either panel independently.

Each television inside a TV panelist's household is equipped with a device that tracks the television's screen activity at every minute interval. The tracked information includes the minute-level timestamp, the television channel, the title of the specific program, and the Nielsen-defined content category associated with that programming. In households with multiple participants, participants are tasked with identifying themselves individually at the onset of a session of television watching, but the data collection is otherwise completely passive. For example, a single row in the television viewership panel represents a single individual initiating a session of watching a television program, including the timestamp at which that session began and the duration spent watching that same program, where that duration is determined by one of four endpoints: the end of the program, the television turning off, the channel being changed, or in the case of co-viewership, a viewer self-identifying that they're no longer watching while someone else in the household is. In rare cases (<0.01% of participants), a participant's total duration of television viewing exceeds the time in which it occurs (e.g., averaging 25 hours of television per day). This overtime viewing can occur due to erroneous self-identification or a single individual keeping multiple television sets on. In these cases, we scale the participant's television viewing to the maximum possible duration (i.e., 24 hours in a day). This scaling is done at the monthly level, based on the number of full days that the participant was active in that month.

For participants in the web browsing panel, tracking software is installed on the user's web browser(s) on the user's primary laptop or desktop computer. A single row of browser panel data includes an initiation timestamp and a duration, where exact URLs are recorded. The duration ends for one of four reasons: the URL is changed, the tab is deactivated, the browser is closed, or the computer ceases to be in an 'awake' mode. As laptops/desktops are presumed individual (versus televisions which are feasibly communal), there is no self-identification process in the browser data collection. Still, if a participant's browser recorded a total duration of activity longer than possible, the participant's web usage is scaled in the same manner as is done with television viewing.

As the basis for all analyses in this paper, we calculate the daily average amount of time each panelist spent consuming news content (defined below as d_{im}) independently in each month. We use demographic weights w_i provided by the Nielsen company to map the sample to the US population. Weights provided for the web browsing panel also incorporate a behavioral component, adjusting slightly to correct for the convenience sample data collection method. Given that both panels are quite large and representative, the inclusion of these weights has only negligible results on our analysis.

2. *Demographic Breakdown of the Sample*

The following four tables provide demographic information on our sample, broken down by month and displaying each demographic variable made available to us. Table S1 describes the TV panel; Table S2 describes the desktop browser panel (web). In the data given to us, gender was coded as a binary variable, so the statistical complement of "Male" in all four tables is "Female." The racial variables provided indicate whether a panelist identified as Asian, Black, or Hispanic, without further racial breakdown.

Month	Total	Male	HS or less	Some Clg.	Clg. Grad	Grad+	Asian	Black	Hisp.	18-24	25-34	35-44	45-54	55+
(2016) 1	100	47.48	35.48	33.37	19.87	11.29	3.80	14.24	14.61	11.11	17.04	16.56	18.39	36.90
2	100	47.42	35.41	33.49	19.80	11.30	3.81	14.24	14.51	11.21	16.97	16.45	18.37	36.99
3	100	47.36	35.23	33.62	19.85	11.30	3.81	14.14	14.51	11.14	16.91	16.44	18.41	37.11
4	100	47.36	35.10	33.65	19.97	11.28	3.84	14.04	14.45	11.05	16.94	16.45	18.46	37.10
5	100	47.26	35.13	33.49	20.05	11.33	3.88	14.07	14.53	11.03	16.90	16.45	18.42	37.20
6	100	47.23	35.12	33.43	20.03	11.42	3.90	14.11	14.63	10.83	16.88	16.54	18.40	37.35
7	100	47.27	35.21	33.37	20.03	11.39	3.85	14.23	14.78	10.76	16.86	16.54	18.38	37.46
8	100	47.25	35.22	33.36	20.01	11.40	3.84	14.17	14.83	10.70	16.89	16.51	18.39	37.51
9	100	47.27	35.05	33.41	20.06	11.47	3.86	14.24	14.83	10.77	16.84	16.52	18.37	37.49
10	100	47.26	34.98	33.44	20.11	11.47	3.84	14.19	14.80	10.92	16.87	16.45	18.33	37.43
11	100	47.26	34.90	33.36	20.20	11.54	3.88	14.26	14.86	10.99	16.87	16.41	18.24	37.49
12	100	47.24	34.74	33.37	20.34	11.56	3.93	14.20	14.88	11.06	16.86	16.39	18.19	37.49
(2017) 1	100	47.26	34.75	33.46	20.23	11.56	3.89	14.22	14.91	11.10	16.83	16.32	18.15	37.60
2	100	47.20	34.64	33.44	20.35	11.57	3.89	14.27	14.95	11.11	16.87	16.23	18.20	37.59
3	100	47.19	34.55	33.43	20.42	11.60	3.90	14.21	14.95	11.12	16.90	16.27	18.12	37.59
4	100	47.24	34.40	33.47	20.44	11.70	3.94	14.28	14.84	11.03	17.02	16.21	18.04	37.70
5	100	47.25	34.36	33.47	20.47	11.71	3.94	14.34	14.85	10.96	17.05	16.25	17.99	37.76
6	100	47.22	34.41	33.41	20.44	11.74	3.86	14.28	14.86	10.78	16.93	16.25	17.99	38.04
7	100	47.16	34.41	33.37	20.49	11.73	3.86	14.33	14.87	10.68	16.82	16.29	18.03	38.18
8	100	47.19	34.56	33.21	20.50	11.73	3.86	14.35	14.99	10.69	16.79	16.34	17.98	38.20
9	100	47.22	34.39	33.22	20.55	11.84	3.84	14.22	14.99	10.62	16.75	16.38	17.86	38.40
10	100	47.25	34.47	33.18	20.57	11.78	3.83	14.37	15.11	10.72	16.80	16.44	17.79	38.26
11	100	47.23	34.34	33.20	20.65	11.80	3.85	14.26	15.26	10.70	16.85	16.39	17.73	38.34
12	100	47.22	34.28	33.21	20.67	11.85	3.90	14.38	15.16	10.73	16.83	16.37	17.64	38.43
(2018) 1	100	47.24	34.38	33.10	20.67	11.84	3.97	14.32	15.31	10.80	16.85	16.36	17.54	38.45
2	100	47.28	34.23	33.12	20.77	11.88	3.99	14.28	15.22	10.74	16.99	16.39	17.47	38.41
3	100	47.24	34.10	33.12	20.78	12.00	3.99	14.41	15.17	10.72	17.03	16.35	17.45	38.45
4	100	47.20	34.02	33.01	20.87	12.10	3.96	14.32	15.14	10.65	17.02	16.44	17.42	38.47
5	100	47.18	33.92	33.02	20.89	12.16	3.93	14.35	15.12	10.50	17.09	16.45	17.41	38.55
6	100	47.11	33.94	32.93	20.89	12.24	3.95	14.38	15.10	10.32	17.05	16.41	17.51	38.71
7	100	47.10	33.95	32.91	20.92	12.22	3.95	14.31	15.18	10.28	16.92	16.47	17.46	38.87
8	100	47.07	33.99	32.91	20.93	12.17	3.96	14.36	15.18	10.22	16.92	16.54	17.37	38.95
9	100	47.00	33.99	32.93	20.85	12.23	3.95	14.33	15.32	10.30	16.89	16.56	17.33	38.92
10	100	47.04	33.94	32.99	20.82	12.26	3.99	14.37	15.39	10.37	16.82	16.53	17.34	38.94
11	100	46.99	33.94	33.08	20.72	12.25	4.07	14.26	15.46	10.48	16.69	16.55	17.29	38.98
12	100	46.99	33.91	33.07	20.81	12.20	4.10	14.23	15.40	10.56	16.66	16.48	17.17	39.12
(2019) 1	100	47.03	33.86	33.13	20.80	12.21	4.10	14.28	15.36	10.53	16.63	16.48	17.14	39.23
2	100	47.00	33.68	33.23	20.75	12.34	4.15	14.29	15.43	10.49	16.63	16.56	17.11	39.22
3	100	47.02	33.81	33.14	20.75	12.31	4.19	14.28	15.53	10.55	16.55	16.57	17.11	39.21
4	100	47.02	33.71	33.12	20.86	12.31	4.25	14.27	15.38	10.44	16.56	16.63	17.13	39.25
5	100	47.00	33.71	33.09	20.90	12.31	4.26	14.42	15.34	10.42	16.50	16.67	17.09	39.30
6	100	47.05	27.41	34.37	24.07	14.16	4.83	13.55	14.90	12.15	19.88	19.24	18.10	30.63
7	100	47.13	27.60	34.03	24.18	14.19	4.68	13.72	15.05	12.02	19.80	19.33	18.19	30.65
8	100	46.99	33.74	32.88	20.97	12.41	4.06	14.66	15.35	10.11	16.14	16.56	17.17	40.01
9	100	47.06	33.72	32.82	21.04	12.42	4.01	14.76	15.43	10.06	16.03	16.52	17.14	40.25
10	100	47.05	33.65	32.82	21.07	12.46	3.96	14.71	15.57	10.16	15.94	16.50	17.12	40.28
11	100	47.06	33.58	32.85	21.10	12.48	3.96	14.70	15.56	10.25	15.91	16.46	17.02	40.35
12	100	47.02	33.75	32.69	21.08	12.48	3.96	14.66	15.55	10.30	15.84	16.34	16.97	40.55

Table S1. Demographic breakdown of the TV panel, as percent of unique individuals each month.

Month	Total	Male	Grad+	Clg. Grad	HS or Less	Some Clg.	Asian	Black	Hisp.	18-24	25-34	35-44	45-54	55+
(2016) 1	100	55.53	8.82	17.54	33.47	39.86	3.33	14.96	10.45	14.18	20.92	17.06	18.58	29.26
2	100	55.52	8.77	17.46	33.56	39.90	3.33	14.86	10.42	14.14	21.13	17.24	18.60	28.88
3	100	55.54	8.85	17.72	33.46	39.66	3.40	14.96	10.52	14.10	21.18	17.28	18.52	28.92
4	100	55.62	8.94	17.86	33.28	39.63	3.38	15.02	10.57	14.21	21.03	17.43	18.44	28.90
5	100	55.64	9.04	18.08	32.89	39.68	3.40	14.98	10.48	14.05	21.15	17.40	18.43	28.97
6	100	55.63	9.15	18.26	32.84	39.46	3.41	15.03	10.47	13.98	21.12	17.45	18.38	29.07
7	100	55.75	9.18	18.24	32.72	39.56	3.41	15.20	10.68	14.11	21.05	17.30	18.29	29.24
8	100	55.78	9.19	18.31	32.76	39.45	3.43	15.44	10.66	14.35	21.16	17.33	18.36	28.79
9	100	55.68	9.30	18.50	32.51	39.41	3.41	15.53	10.72	14.12	21.13	17.36	18.44	28.95
10	100	55.68	9.27	18.64	32.42	39.38	3.40	15.30	10.61	14.13	20.97	17.22	18.37	29.31
11	100	55.71	9.23	18.71	32.38	39.40	3.40	15.29	10.57	14.19	21.10	17.21	18.34	29.17
12	100	55.81	9.21	18.65	32.29	39.57	3.39	15.38	10.54	14.41	21.12	17.25	18.31	28.92
(2017) 1	100	55.94	9.24	18.59	32.31	39.59	3.31	15.52	10.58	14.39	20.90	17.20	18.27	29.23
2	100	55.63	9.17	18.55	32.42	39.58	3.41	15.57	10.82	14.67	21.06	17.27	18.31	28.70
3	100	55.90	9.40	18.87	31.90	39.57	3.45	15.54	10.84	14.59	20.69	17.25	18.25	29.23
4	100	55.91	9.54	19.07	31.52	39.63	3.47	15.51	10.80	14.43	20.63	17.19	18.24	29.50
5	100	55.90	9.64	19.24	31.26	39.62	3.51	15.56	10.90	14.44	20.51	17.16	18.21	29.67
6	100	55.82	9.69	19.48	31.11	39.49	3.57	15.41	10.92	14.32	20.46	17.02	18.16	30.04
7	100	55.95	9.88	19.84	30.64	39.43	3.63	15.58	10.80	14.42	20.57	16.97	18.25	29.79
8	100	61.96	11.20	21.69	25.39	41.72	3.53	15.29	10.84	13.10	20.76	17.42	18.04	30.67
9	100	55.62	10.30	20.57	29.92	39.01	4.00	15.19	11.03	15.32	21.03	16.92	17.91	28.82
10	100	55.64	10.34	20.63	29.85	38.99	3.96	15.21	11.04	15.32	21.06	16.87	17.80	28.95
11	100	55.56	10.37	20.69	29.77	39.01	4.00	15.23	11.33	15.37	21.42	16.90	17.64	28.67
12	100	55.43	10.43	20.70	29.80	38.91	4.04	15.37	11.31	15.61	21.38	16.91	17.59	28.50
(2018) 1	100	55.53	10.35	20.71	29.88	38.91	3.96	15.49	11.30	15.46	21.16	16.92	17.65	28.80
2	100	55.47	10.33	20.77	29.77	38.99	3.95	15.52	11.60	15.91	21.21	16.72	17.60	28.56
3	100	55.65	10.34	20.88	29.62	39.00	3.92	15.61	11.60	16.02	20.85	16.73	17.55	28.85
4	100	55.66	10.40	20.96	29.52	38.97	4.01	15.61	11.59	16.14	20.85	16.78	17.52	28.71
5	100	55.75	10.43	20.97	29.54	38.91	4.00	15.71	11.57	16.31	20.77	16.75	17.40	28.75
6	100	55.79	10.48	21.00	29.54	38.85	3.99	15.76	11.72	16.14	20.82	16.63	17.48	28.94
7	100	55.88	10.58	21.05	29.52	38.72	4.00	15.82	11.76	16.12	20.80	16.58	17.37	29.14
8	100	55.88	10.66	21.05	29.34	38.83	4.02	15.62	11.94	16.15	20.91	16.48	17.39	29.06
9	100	55.95	10.71	21.08	29.29	38.81	4.06	15.56	12.04	16.10	20.82	16.57	17.34	29.18
10	100	55.98	10.41	20.75	29.55	39.19	3.91	15.63	12.02	16.46	20.43	16.50	17.30	29.31
11	100	56.04	10.23	20.57	29.88	39.22	3.92	15.64	11.96	16.30	20.48	16.49	17.24	29.49
12	100	56.05	10.06	20.51	29.76	39.55	3.79	15.57	12.03	16.49	20.35	16.35	17.20	29.62
(2019) 1	100	61.14	10.85	21.35	25.47	42.33	3.52	16.00	11.36	15.15	20.13	17.03	17.05	30.64
2	100	61.34	10.93	21.46	25.37	42.23	3.52	15.87	11.78	15.40	19.96	17.09	16.99	30.56
3	100	61.48	11.31	21.88	24.62	42.19	3.68	15.71	11.76	15.77	20.05	16.83	16.96	30.40
4	100	61.21	11.23	21.74	24.99	42.05	3.69	15.61	11.77	15.37	19.83	16.55	17.02	31.23
5	100	61.02	11.20	21.54	25.06	42.20	3.74	15.66	11.85	15.56	19.60	16.51	17.17	31.16
6	100	61.31	11.04	21.61	25.27	42.08	3.77	15.95	11.75	15.48	19.46	16.56	17.16	31.34
7	100	61.26	10.99	21.61	25.41	41.99	3.69	16.14	11.92	15.20	19.65	16.66	16.97	31.52
8	100	61.02	11.06	21.67	25.26	42.01	3.64	16.04	12.14	15.06	19.56	16.87	16.95	31.55
9	100	61.14	11.11	21.66	25.19	42.04	3.69	16.02	12.23	14.83	19.44	16.95	16.92	31.86
10	100	61.18	11.05	21.72	25.39	41.84	3.57	16.24	12.07	14.58	19.49	17.02	17.04	31.87
11	100	61.01	11.05	21.67	25.33	41.95	3.57	16.14	12.04	14.37	19.28	17.00	17.03	32.32
12	100	61.04	11.01	21.46	25.51	42.02	3.57	16.08	12.01	14.31	19.04	17.11	16.99	32.54

Table S2. Demographic breakdown of the desktop browser panel, as percent of unique individuals each month.

3. TV News Operationalization

To identify news programming on television, we first apply the broadest 'news' category definition maintained by the Nielsen Company. This includes programming focused on political information, political commentary, sports-related information, weather information, and celebrity gossip. We remove all television programming that is explicitly or exclusively non-political. This definition keeps variety programming such as *The TODAY Show* and *Good Morning America* in our news dataset, as such programs often discuss current affairs, while avoiding biasing our results with wholly non-political news. We then aggregate television news programs into intuitive discrete categories used to build the archetypes in Figures 3 and 4. First, we identify hundreds of 'broadcast affiliate' stations which present news programming from one of seven English-language broadcast networks: *ABC*, *CBS*, *CW*, *Fox*, *ION*, *NBC*, and *PBS*. The "Big Three" dominate this set: ABC, CBS, and NBC. Note that the Fox Affiliates and Fox broadcast are not the same as Fox News, a cable news station. The similarity of news content from these seven sources leads us to combine their programming into two categories: 'hard broadcast news' and 'soft broadcast news'—that is, programming entirely focused on politics and current affairs (e.g., *NBC Nightly News*) versus variety programming with some amount of regular political content (e.g., *Good Morning America*).

Next, news programming from cable stations with very low news viewership (e.g., with only one weekly program coded as 'news,' or very low ratings) were aggregated into a single news category (e.g., *BBC America, Al Jazeera America*). We aggregate all Spanish-language news programming into a single category. This includes news programming from CNN Español, Estrella, Telemundo, and Univision. While the archetypal audience of Spanish-language TV news is quite concentrated (i.e., these viewers watch little to no English-language news, as shown in Figure 3), this does not imply homogeneity of bias in Spanish language news or speak to the average partisan bias of Spanish-language news as a whole. Lastly, we relabel news programming from 'Fox Business' as being from the Fox News Channel due to programming similarity. The remaining cable channels are CNN, Fox News, and MSNBC, which are also used in Figures 1 and 2. This source-categorization schema is visualized Table S3.

**Group**	**Examples**	**Partisan Bias**
MSNBC	*MSNBC*	Yes
CNN	*CNN*	By lenient definition
Spanish Language	All news programs on e.g., *Telemundo, Univision, WAPA*	No
Other Cable	All news programs on e.g., *Al Jazeera America, BBC, BET, HLN*	No
'Hard Broadcast'	All hard news programs on e.g., *ABC,CBS, FOX, NBC*	No
'Soft Broadcast'	All soft news programs on e.g., *ABC,CBS, FOX, NBC*	No
Fox News	*Fox News Channel* and *Fox Business Channel*	Yes

Table S3. Grouping television news programming. Blue to red shading indicates left to right partisan slant. Note that *Fox Affiliates*, a broadcast network, is distinct from *Fox News Channel* and *Fox Business Channel*.

4. *Web News Operationalization*

To classify web-browsing behavior as 'news consumption' or not, we first apply Nielsen's categorization schema at the domain level. News 'domain' refers to the top-level URL feature of a news website. E.g., the domain for any New York Times article hosted by the New York Times' own website is *nytimes.com*. More than three thousand unique web domains (out of all web domains accessed by participants) were categorized by Nielsen as being news providers. These websites include well-known publishers (e.g., *nytimes.com*, *huffpo.com),* cable-news online (e.g., *Foxnews.com*, *cnn.com*), broadcast affiliates online (e.g., *abc7.com*, *nbcLosAngeles.com*), small local publications (e.g., *lowellsun.com*, *omaha.com*), specific political outlets (e.g., *redstate.com*, *commondreams.org*), government institutions (e.g., *state.gov*, *senate.gov*), news aggregators (e.g, *news.aol.com*, *news.google.com*), and non-political news coverage such as sports, gossip, finance, weather, and tech news. We manually removed news sites that are explicitly or exclusively non-political in their primary content. We note also that our strategy automatically captures news websites that were arrived at *via* social media, but not political content consumed directly *on* social media or video streaming websites. We know from prior research that approximately 6.4% of news consumption occurs on smartphones, which we do not analyze here (28). (This percentage is computed using Table S6 in the *Supplementary Materials* of Allen et al. (28)). Thus, even while restricting our study to the web-browsing of desktop/laptop browsers, we estimate that we still capture the vast majority of news consumption.

To assign partisan bias labels to news domains, we draw on audience behavior. Specifically, we make use of (18), which provides a list of domains scored between 0 (extreme left) and 1 (extreme right) according to large-scale sharing behavior on Twitter. The scores are based on proportions of left- and right-leaning Twitter users who shared links from each domain during a sampling period in 2018. The intersection of this set and the news domains encountered in the Nielsen panel is 1,718 domains, which together account for more than 95% of online news consumption time in our panel. For our analysis, we reduce the scoring system provided by (18) to an ordinal ranking system. The source categorization schema is shown in Table S4.

**Group**	**Examples**	**Partisan Bias**
Furthest :eft	*Dailykos.com, msnbc.com, slate.com, vox.com*	Yes
Left	*npr.org, pbs.org, theguardian.com, theatlantic.com*	By lenient definition
Centrist News	*cnn.com, nytimes.com, usatoday.com, wapo.com*	No
Portals/Aggregators	*news.aol.com, news.msn.com, news.yahoo.com*	No
Right	*Dailycaller.com, foxnews.com, realclearpolitics.com*	By lenient definition
Furthest Right	*Breitbart.com, drudgereport.com, theblaze.com*	Yes

Table S4: Grouping news websites. Blue to red shading indicates left to right partisan slant.

Figure S1 is a histogram showing the resulting distribution of unique websites across a spectrum of bias, as well as locations of bias thresholds used in the main paper. Note that this histogram shows the count of unique domains at each bias score level, not the frequency of visits or visitors. We reduce the continuous scoring system visualized here to an ordinal ranking system.

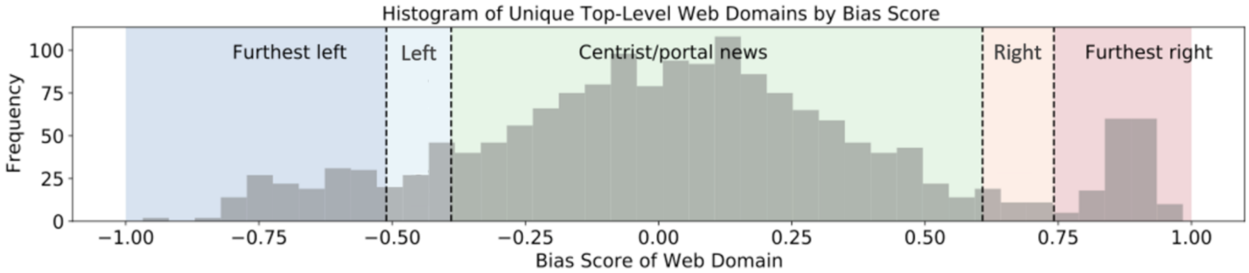

Figure S1: Partisan ranking of news domains visited by panelists. Blue to red shading indicates left to right partisan slant.

We recognize the theoretical limitations of audience-based bias rankings. In the absence of convincingly objective content-based rankings of the partisan bias of web domains, we offer a comparison of our domain ranking against another established source in the field, with lower coverage. Partisan rankings for 143 domains compiled by Eady et al. (16) were generated by mapping the self-reported partisan placement of 1,496 Twitter users to ~1.2 billion tweets. To compare the partisan domain rankings of Robertson et al. (18) with those of Eady et al. (16), we identify the intersection between these two sets of domains while preserving their respective orders, and then calculate the Pearson correlation coefficient between them. The Pearson correlation coefficient is a nonparametric bivariate summary statistic for comparing ordinal lists. A score of -1 implies a perfect negative correlation between lists, while a score of +1 implies a perfect positive correlation between lists. Our comparison yielded a correlation coefficient of 0.929, suggesting an extremely clear correlation between the two. While Eady et al. (16) followed similar methods as Robertson et al. (18), we feel that the similarity in their results speaks to the fidelity of web domains' partisan ranking, robust to the time, scale, and manner of data collection.

To prove this point further, we also compare our domain ranking against those established by Bakshy et al. (2015)(8). These scores were based on the URL sharing patterns of Facebook users who identified with a political party, seven years prior to the current analysis. This Facebook-derived set includes 500 domains. We again find the intersection between this set and our preferred set, preserve the rankings of either source, and calculate the Pearson correlation coefficient between those rankings. This comparison yielded a correlation coefficient of 0.962, a remarkably high correlation. Hence, our chosen domain-ranking system is not only corroborated by a separate analysis on the same data source (versus Eady et al., (2018) (11)), but also by an analysis using an *alternative* data source, Facebook.

5. *Delineating bias in news content*

The prior section explained the creation of ordinal rankings of online news partisanship. This section now describes how we identify whole categories of online news using this ranking, along with our analogous approach to sorting television news programming.

Theoretically, partisan bias is a continuous variable, but one that is essentially impossible to quantify precisely in a universally satisfying way; partisan-ideological sorting is imperfect, positions change, and interpretations are not concrete. However, as the focus of this paper is the extent to which individuals are surrounded by clearly partisan content, we leave genuine partisan extremity as a latent variable, and classify news content in discrete terms: news content is either partisan or not, and either left or right. To do this, we first identify familiar online news content that is broadly reputed to present partisan bias, and set that content as the threshold between 'partisan' and 'not partisan.' Among news sites, we do this based on the ordinal ranking of domains established by Robertson et al. (18). For television news, we do not try to map the various news programs to a continuum, or to systematically characterize subtle ideological differences between them. We instead utilize the conventional understanding that *Fox News* and *Fox Business* are furthest right among large-scale television news networks, *MSNBC* is furthest left, and the rest of the channels, including all of the major broadcast networks, adhere more closely to a centrist approach. The one major television news source we concede as having arguable status is the remaining large-scale cable news network, *CNN*. We demonstrate the effect of including *CNN* as a left or centrist news network in Figure 1 Panel A and B respectively. Endogenous clustering of television news program viewership recreated the major conventional groups: major cable channels, hard broadcast, soft broadcast.

In contrast to television news, news websites are not aggregated by channels, and so bias categorization is done at the level of the individual news website. On the left, we identify *slate.com* as the stringent boundary for the left, and *theguardian.com* as the lenient boundary for the left. On the right, we identify *breitbart.com* as the stringent boundary for the right, and *Foxnews.com* as the lenient boundary for the right. Hence, any news websites with a bias ranking beyond these boundaries are considered to be partisan.

Importantly for our categorization schema, television news stations with analogous web domains (e.g., *CNN* & *cnn.com*, *MSNBC* & *msnbc.com*, *Fox News* & *Foxnews.com*) do not serve as a direct bias mapping from online to television news, and they are treated distinctly in our analysis. For example, we choose to set *Foxnews.com* as our lenient boundary for right-biased news domains. On television, Fox News dominates right-leaning news programming. This should not be interpreted to mean that all rightward-biased television news programming is only as biased as the most leniently right-biased news domain we include, *Foxnews.com*. Given that our bias rankings for web domains were based on audience measures, the long thin tail of right-leaning web domains nudges *Foxnews.com* empirically leftward. Conceptually, this same rank ordering would apply *within* Fox News, which is not crowded-out with small competitors, such that the most left-leaning Fox News program serves as our boundary for rightwardly-biased news, and a long tail of *Fox News* programs exists to the right of that boundary.

6. Demographic breakdown of partisan audience segregation, in absolute count

Table S5 shows partisan segregation sizes for various demographic groups, calculated per month and then averaged across the four-year span of our data. This is identical to Table 1 in the main text but uses absolute counts rather than percentages. Absolute sizes are estimated using demographic weights provided by Nielsen. Intra-demographic percentages are estimated according to the population size of the corresponding demographic in the United States. We source all population estimates from the most recent US Census figures.

Partisan Segregated TV News Audience Sizes, by Number of Adults

Bias	Left				Right	
Bias labelling strategy	Lenient		Strict		(Fox News)	
Diet bias proportion	0.50	0.75	0.50	0.75	0.50	0.75
All adults	22,174,015	14,982,556	9,094,563	5,330,368	21,434,808	16,470,352
White	13,967,089	9,298,889	6,296,336	3,751,914	19,530,279	15,094,126
Non-white	8,206,926	5,683,666	2,798,228	1,578,454	1,904,529	1,376,226
<25yo	1,351,091	996,913	433,865	289,825	1,094,675	865,366
55yo+	11,474,368	7,489,546	5,592,190	3,166,739	13,484,509	10,508,624
HS diploma or less	4,141,518	2,595,265	1,527,434	845,758	5,510,654	4,153,253
Some college	5,961,569	3,932,564	2,446,940	1,400,498	7,007,688	5,405,734
College grad	5,769,066	3,958,529	2,341,781	1,396,492	5,122,405	3,941,164
Post-graduate	5,113,451	3,618,390	2,390,140	1,427,664	2,813,791	2,189,966

Partisan Segregated Desktop News Audience Sizes, by Number of Adults

Bias	Left				Right			
Bias labelling strategy	Lenient		Strict		Lenient		Strict	
Diet bias proportion	0.50	0.75	0.50	0.75	0.50	0.75	0.50	0.75
All adults	4,886,139	2,855,422	2,364,091	1,296,533	5,816,393	3,638,348	2,216,877	1,152,102
White	3,365,013	1,895,613	1,655,452	893,916	5,208,768	3,270,052	2,037,899	1,055,361
Non-white	1,521,126	959,809	708,638	402,616	607,625	368,296	178,978	96,740
<25yo	625,383	402,824	254,756	152,217	195,308	121,016	69,322	40,211
55yo+	1,721,404	980,038	907,596	478,032	3,269,978	2,067,999	1,421,068	740,677
HS diploma or less	560,954	369,455	263,844	156,373	987,981	660,528	419,115	239,103
Some college	1,519,832	909,246	761,672	423,749	2,098,880	1,348,744	842,037	449,434
College grad	1,308,969	734,691	670,214	354,176	1,623,495	1,005,739	592,136	296,058
Post-graduate	934,284	479,145	444,552	228,752	936,670	518,931	305,052	133,540

Table S5. Partisan segregation size estimates under multiple parameterizations and for various demographic cohorts.

7. Setting and varying the minimum threshold of time spent consuming news

In the main text, identification as a news consumer is based on the amount of time in a month that an individual spends consuming news. In the lenient operationalization common in the literature, anybody who watches any amount of television news in a given month is considered a news consumer, and likewise for desktop news consumption. This approach raises three practical concerns. First, an individual consuming a very small duration of news has less opportunity to diversify their news diet, and is thus statistically biased toward being counted as partisan segregated. Second, an individual consuming a very small duration of news is more likely to have accidentally encountered the news content (i.e., by channel surfing or following a link just once) and thus is not actually an active news consumer. Third, individuals who only consume a very short duration of news are not the core population of politically minded individuals with dangerously skewed news diets. As such, we set our stringent bound of television

news consumption at thirty minutes per month, which represents a single thirty-minute news program in a month, or roughly one minute of television news per day. For online news consumption, we set this threshold to two minutes per month. This monthly threshold is proportionate to our thirty-minute monthly threshold for television consumption, based on the average amount of time that Americans spend consuming news from either platform. In Figure S2, Figure 1 from the main text is recreated without setting minimum thresholds of news consumption. That is, anybody who consumes just a moment of news online or on TV is counted as a news consumer, and hence may be counted as experiencing partisan segregation.

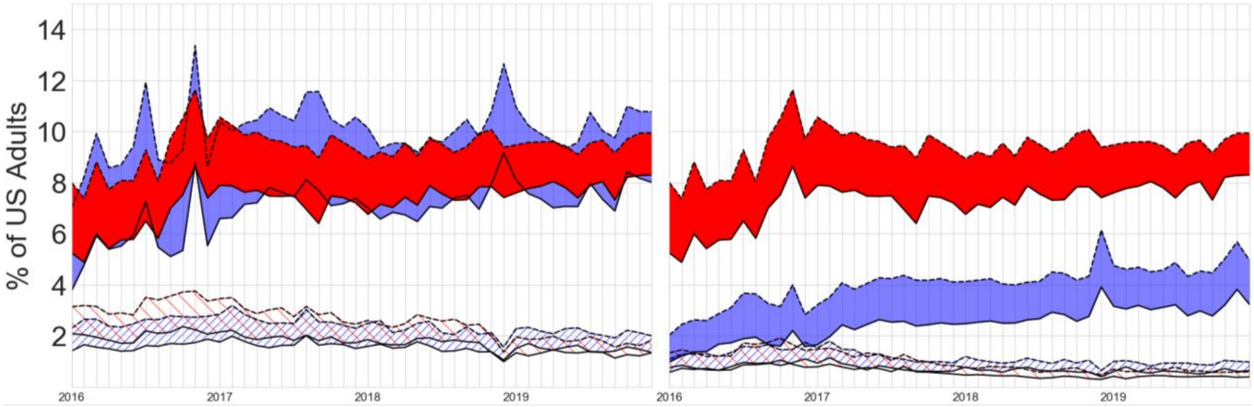

Figure S2. Recreation of Figure 1 without setting minimum thresholds of news consumption (parameter 3). The monthly percent of Americans experiencing partisan segregation via TV or web news. Bounds (dotted and dashed lines) represent strict or lenient values of the percent of intra-individual news diets that must be partisan for partisan segregation (parameter 1). In Panel A on the left, websites more partisan than *TheGuardian.com* [*FoxNews.com*] are counted as left [right]. In stricter Panel B on the right, partisan content bounds are *Slate.com* [*Breitbart.com*] on the left [right]. CNN is counted as left-leaning in Panel A.

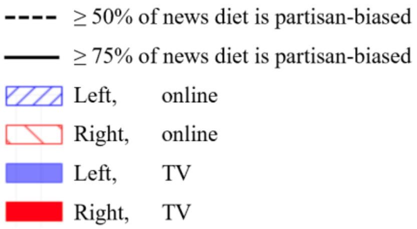

8. *Partisan Segregation Experience Sessions: Survival Analysis*

Figure 2 estimates the probability of a partisan segregation experience session lasting longer than *t* months, for either platform and either partisanship. For this we use the *lifetimes* Python package (57) to conduct a survival analysis. As described in the Materials and Methods section in the main text, we apply the Kaplan-Meier estimator (43),

$$\hat{P}(t) = \prod_{m:t_m \leq t} (1 - \frac{d_m}{n_m})$$

where *m* indexes months from the start of a session of partisan segregation (i.e., $m = 1$) to *t*, n_m is the number of audience members experiencing partisan segregation still known to be active by month *m*, and d_m is the number of individuals

who are no longer experiencing partisan segregation in month *m*. Months are standardized according to the start of each partisan segregation session; *m*=1 can refer to any calendar month in our four-year panel period. Individual panelists may have multiple membership sessions throughout their participation in the panel, separated by one or more months. By design, the Kaplan-Meier estimator accounts for right-censoring, which is crucial for analyzing behavior within the rotating panels. Left-censoring, which is theoretically symmetrical to right-censoring in our panel, was not directly addressed in this paper, but may be an avenue for future methodological research. The authors considered removing panelists whose first panel month was also the start of a partisan segregation session, as we cannot determine their news diets in preceding months. However, doing so would bias our results by targeting individuals with longer or more frequent partisan segregation sessions, as these individuals would be more likely to face this leftward censoring by definition. Hence, in these cases, the panelist's first month of panel membership is coded as *m*=1 for partisan segregation by assumption. Bootstrap-based confidence intervals calculated for the point estimates in Figure 2 were small enough to be indiscernible. Table S6 provides the data underlying Figure 2, as well as the same values expressed as the percentage of Americans.

Multi-Month Survival Rates of Partisan News Diets

	Likelihood of having same news diet				Expressed as percentage of American adults			
	TV		Web		TV		Web	
Months since first partisan news diet	Left	Right	Left	Right	Left	Right	Left	Right
0	1.00	1.00	1.00	1.00	8.71	8.42	1.92	2.29
1	0.46	0.54	0.21	0.29	4.05	4.57	0.40	0.67
2	0.33	0.42	0.09	0.17	2.87	3.57	0.16	0.38
3	0.27	0.36	0.05	0.12	2.35	3.05	0.09	0.27
4	0.23	0.33	0.03	0.09	2.05	2.75	0.06	0.21
5	0.21	0.30	0.02	0.07	1.84	2.54	0.05	0.17
6	0.19	0.28	0.02	0.06	1.69	2.38	0.04	0.14
7	0.18	0.27	0.01	0.05	1.57	2.24	0.03	0.12
8	0.17	0.25	0.01	0.05	1.48	2.13	0.02	0.11
9	0.16	0.24	0.01	0.04	1.40	2.04	0.02	0.10
10	0.15	0.23	0.01	0.04	1.33	1.96	0.02	0.09
11	0.14	0.22	0.01	0.03	1.26	1.88	0.01	0.08
12	0.14	0.22	0.01	0.03	1.22	1.82	0.01	0.07

Table S6. Survival analysis of news audiences with left-biased or right-biased news diets via TV and online. The four columns on the left show likelihood estimates underlying Figure 2 in the main text. The four columns on the right reframe these likelihood scores in terms of percent of Americans. E.g., in expectation, 14% of all left-biased TV news consumers maintain a left-biased news diet for 12 consecutive months. Based on the number of Americans with left-biased TV news diets, this is roughly equivalent to 1.22% of Americans. As with Figure 2 in the main text, Table S6 follows a lenient approach to parameter 1 (news diet composition) and parameter 2 (news partisanship).

9. Partisan Segregation Churn & Time Scales

The individual-level analysis in Figure 2 is distinct from, but closely related to, the aggregate-level phenomenon of individuals rotating into and out of experiencing partisan segregation across time periods. To measure the aggregate level of churn of partisan segregation, along with the robustness of our findings to aggregation units larger than one month, we calculate the average *remain rates* of partisan segregation from one time period to the next

using 4 aggregation units: 1 month (as used throughout the main text), 2 months, 6 months, and 12 months. Figure S3 shows the results of the procedure described below.

First, to calculate the average size of each partisan-segregated audience for a given range of months *r* (where *r* = 1, 2, 6, or 12), call *S* the set of all stretches of *r* consecutive months in the data. For example, when *r* = 2, *S* = {(Jan 2016, Feb 2016), (Feb 2016, Mar 2016), ..., (Nov 2019, Dec 2019)}. As our data lasts 48 months, ||*S*|| = 48-*r*-1. For each stretch of months s_i, we identify the set of panelists who participated in the panel in *every* month in s_i. Call this set $P_{persistent}$, which is the intersection of {P_i, P_j, P_j+1, ..., P_j+r-1}, where P_j is the set of active panelists in month *j*. Per the design of our raw data, each panelist *i* has a demographic weight w_{im} in each month *m*, so when range *r* > 1 (as in Panels 2, 3, & 4 of Figure 2), the demographic weights of $P_{persistent}$ must be combined across all months in s_i. For each month *m*, we update all the panelist weights to account for the disinclusion of panelists who do not appear in $P_{persistent}$. The weight for panelist *i* in month *m* is reassigned as:

$$w_{im} = w_{im} \times \sum (w_m) / \sum (w_m | i \in P_{persistent})$$

Since the original monthly weights all map to a single static description of the U.S. population, the resulting monthly w_{im} for $P_{persistent}$ are theoretically identical, with minor variation across months in s_i based on the demographic particulars of which panelists joined or exited the panel during s_i. To address this small monthly variation, which is assumed to be random, we take the simple average of w_{im} of $P_{persistent}$ across all months in s_i. We then calculate the number of Americans who experience partisan segregation for each time window s_i in *S*, each of which is *r* months long. The news consumption time thresholds used for pruning minimal news consumers are updated according to *r*, i.e., (*r* × 30 minutes) and (*r* × 2 minutes) for television and web respectively. Lastly, we compute the *y*-axis values of Figure S3 by averaging the partisan-segregated audience sizes across all (48-*r*-1) time periods in *S*, for $r \in \{1, 2, 6, 12\}$.

Second, we calculate the remain rate for each of the four types of partisan segregation at each range *r*. Using the above notation, the remain rate is the share of individuals experiencing partisan segregation in time period s_i who remained in the same partisan segregated audience in adjacent time period $s_{(i+r)}$. For example, if *r* = *2*, and s_i = {Jan 2016, Feb 2016}, then $s_{(i+r)}$ = {Mar 2016, Apr 2016}. We restrict analysis to $P_{persistent}$, consisting of all panelists who were active panel members in all months of s_i and $s_{(i+r)}$. As remain rate is a feature of s_i, not $s_{(i+r)}$, we reweight panelists according to s_i only. Similarly, the number of s_i for which we can calculate remain rate is capped by the existence of a subsequent $s_{(i+r)}$ in our 48 month panel, i.e., the remain rate is calculated for 48-2*r*+1 time periods. Lastly, we compute the *x*-axis values of Figure S2 by averaging the partisan-segregated audience sizes across all (48-2*r*-1) time periods, for $r \in \{1, 2, 6, 12\}$. Surprisingly, only a little more than half of right-leaning online partisan-segregated audience members actually remain experiencing their respective partisan segregation by the following month, and only a minority of left-leaning online partisan-segregated audience members remain by the following month. This monthly churn is obscured by aggregated data on online partisan segregation. In contrast, a clear majority of TV news echo-chamber members maintain their partisan-skewed news diet over time.

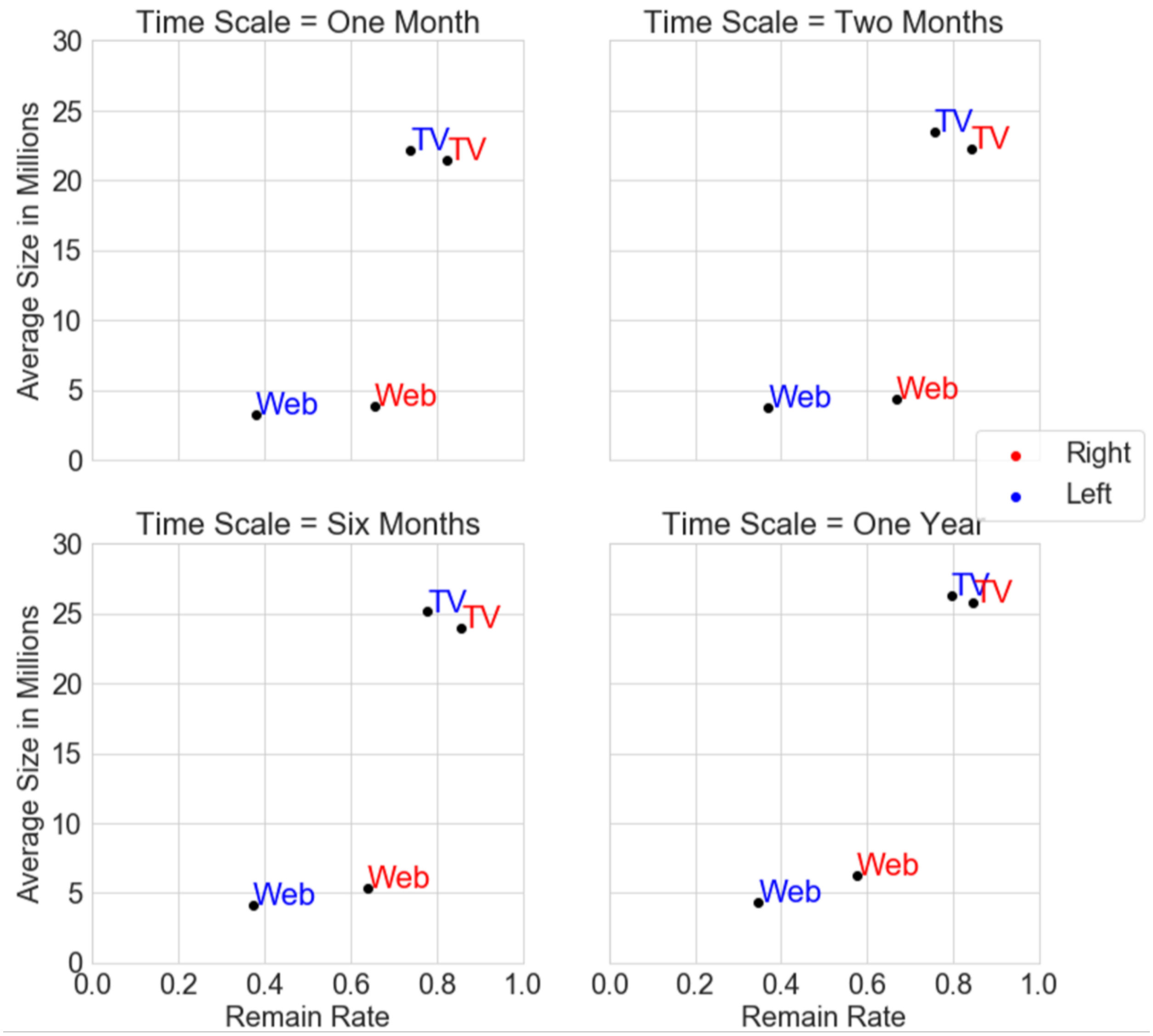

Figure S3. Partisan-segregated audience remain rates. Churn and size calculated as averages across all months (top left, n = 48), all two-month stretches (top right, n = 47), all six month stretches (bottom left, n = 43) and all year-long stretches (bottom right, n = 37).

10. Identifying Archetypes of News consumption

To identify *archetypes* of news consumption via either web or TV, the first step is to identify categories of news on either platform, rather than clustering the vast number of television programs and websites. Television news programming was sorted into seven categories following from their natural grouping into channels, as laid out in Table S3. For desktop news consumption, we create these categories as follows. First, we treat portal websites as a singular category (e.g., yahoo.com, news.google.com) due to their structural similarity and similar mainstream appeal. We then divide the remaining websites into five categories (furthest-left, left, mainstream, right, furthest-right) based on the websites previously used to bound our lenient and stringent definitions of partisan bias.

Independently for either panel, these content categories are treated as independent dimensions for each panelist, measured by minutes spent consuming each category of content in an average month. Thus, each television panelist is assigned a seven-dimensional consumption vector, and a six-dimensional consumption vector is assigned to each desktop panelist. In either panel, we find the cosine similarity between every pair of panelists. This process creates two complete graphs, one for each panel, in which the nodes represent panelists and the edges that connect them are weighted according to the pairwise similarity of news diets. The two complete graphs are then pruned by removing any edges weighted below 0.97, while the remaining edges become unweighted. Outcomes are robust to moderate variations in threshold selection. Finally, in either graph, we run a Louvain community detection algorithm to identify communities of similar news consumers (including the large group of non-news consumers, omitted from Figure 3) (45). The Louvain method was chosen over other community detection methods primarily due to the lack of hyperparameter tuning needed. Moreover, Louvain provided the most intelligible results, based on auditing by the authors. By averaging together the news consumption vectors of panelists in each community, we determined the finite set of news consumption archetypes shown in Figure 3.

11. Growth and shrinkage of archetype popularity over time

In Figure S4, we illustrate the dynamics of all TV news consumption archetypes between 2016 and 2019. As the popularity of broadcast news has decreased over this period, the size of cable news audiences (for CNN, Fox News, MSNBC) has risen. Figure S4 also demonstrates the large increase in the share of Americans who do not watch news on television; this has been on the rise since a local minimum in the 2016 election. In purple, we show the number of Americans who are not television news consumers but have access to television, based on the Nielsen company's estimation of the market, and empirically, individuals' inclusion in the Nielsen panel data. Figure S5 illustrates change in the web archetypes, using the same format.

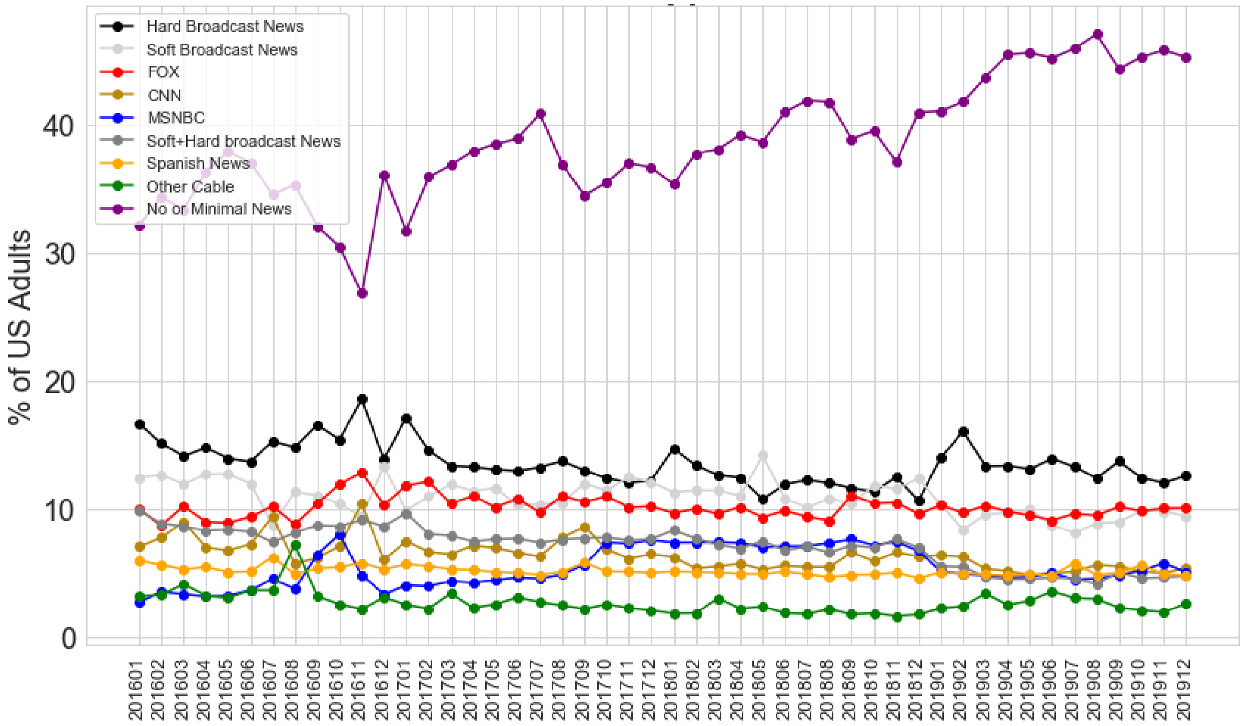

Figure S4. The estimated size of each TV news consumption archetype, as a portion of Americans, 2016-2019. All panelists in the data are assigned to a single archetype, or the "No or Minimal News" group. In purple, we show the difference between the entire adult U.S. population and the number of news consumers identified in our data.

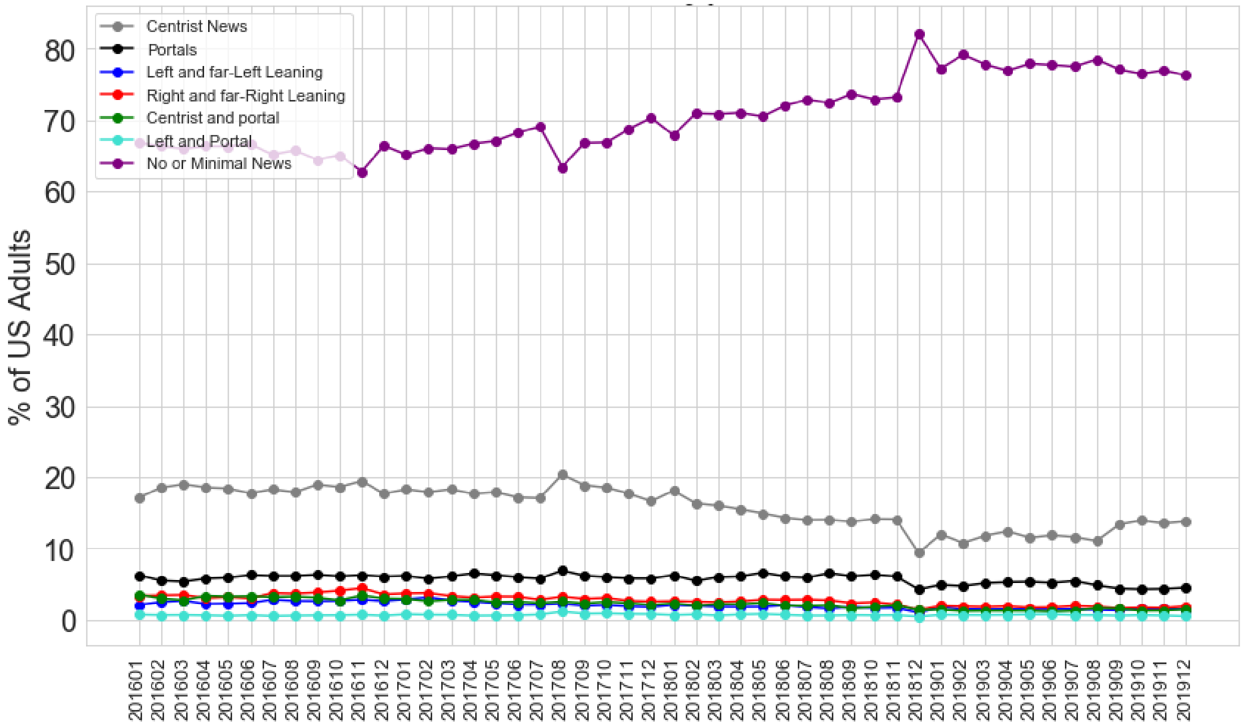

Figure S5. The estimated size of each web news consumption archetype, as a portion of Americans, 2016-2019. All panelists in the data are assigned to a single archetype, or the "No or Minimal News" group. In purple, we show the difference between the entire adult U.S. population and the number of news consumers identified in our data.

Figure 4 in the main text illustrates the flow between archetypes of television news consumption over the four-year duration of our panel. In Figure S6 below, we create the same plot for web consumption archetypes. As with television news consumption, we see that the strongest attractor is a move away from consuming news. Other net dynamics are relatively small, suggesting that no archetype has gained or lost large numbers of Americans. This low net flow—which should not be confused for stability or lack of exchange between archetypes—is in line with the findings of Figures 2 and S3: individuals who exhibit skewed online news diets often show different news diets by the next month. That is, the temporal instability of online partisan segregation and web news archetypes is noisy enough to drown out possible macro-trends in Americans' news diets.

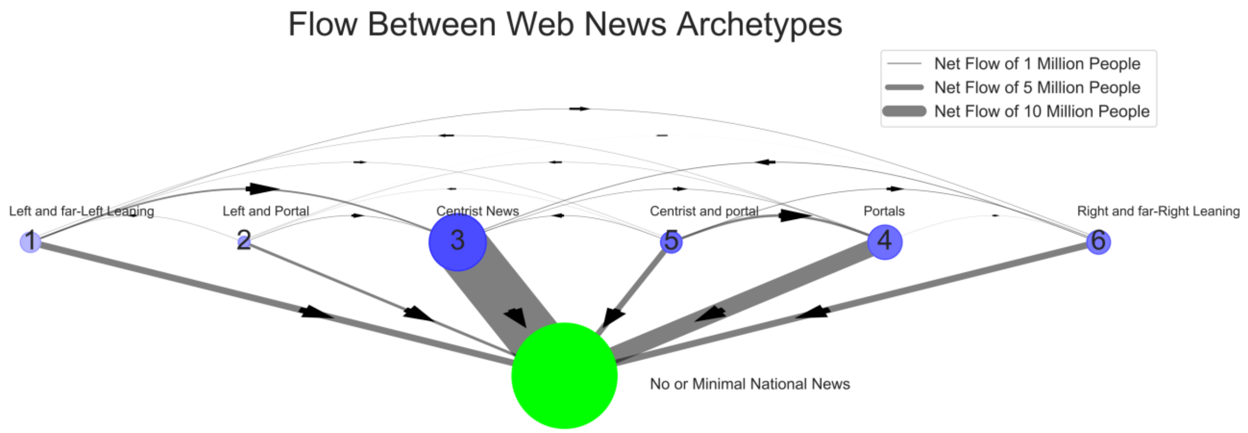

Figure S6: The "net flow" of people between pairs of nine television news archetypes. These are the eight television news archetypes seen in Figure 3 Panel 2, each labeled according to the category of news most prominently consumed, and a 9th group of people who are exposed to less than 2 minutes per news in a month, over the 4 years of analysis. Net flow represents the direction and magnitude of turnover between a pair of archetypes. Specifically, if we let A_i^k be the set of people in archetype i during month k, the "net flow" between archetypes i and j is defined as the absolute value of the expression $\left|A_i^k - A_j^{k+1}\right| - \left|A_j^k - A_i^{k+1}\right|$ summed over all pairs of months (k,$k+1$). The direction of the net flow, signified by the arrows, points toward group j if net flow, before absolute value, is positive, and toward group i if it is negative. We do not show net flows of less than 1M people. Node diameter corresponds to the size of the population of the archetypal cluster averaged over all months. Green signifies that an archetype has experienced net inflow, while blue signifies net outflow, with alpha levels corresponding to the scale of net in[out] flow.

REFERENCES AND NOTES

1. J. A. Tucker, Y. Theocharis, M. E. Roberts, P. Barberá, From liberation to turmoil: Social media and democracy. *J. Democr.* **28**, 46–59 (2017).

2. E. Shearer, *News Use Across Social Media Platforms 2017* (Pew Research Center's Journalism Project, 2017).

3. M. Wojcieszak, E. Menchen-Trevino, J. F. F. Goncalves, B. Weeks, Avenues to news and diverse news exposure online: Comparing direct navigation, social media, news aggregators, search queries, and article hyperlinks. *Int. J. Press Polit.* 10.1177/19401612211009160 (2021).

4. L. Terren, R. Borge, Echo chambers on social media: A systematic review of the literature. *Rev. Commun. Res.* **9**, 99–118 (2021).

5. C. R. Sunstein, *#Republic: Divided Democracy in the Age of Social Media* (Princeton Univ. Press, 2018).

6. E. Pariser, *The Filter Bubble: How the New Personalized Web Is Changing What We Read and How We Think* (Penguin, 2011).

7. C. R. Sunstein, Democracy and filtering. *Commun. ACM* **47**, 57–59 (2004).

8. M. S. Levendusky, How does local TV news change viewers' attitudes? The case of Sinclair broadcasting. *Polit. Commun.* **39**, 23–38 (2021).

9. N. J. Stroud, Polarization and partisan selective exposure. *J. Commun.* **60**, 556–576 (2010).

10. L. Muradova, Seeing the other side? Perspective-taking and reflective political judgements in interpersonal deliberation. *Polit. Stud.* **69**, 644–664 (2020).

11. Y. Shmargad, S. Klar, How Partisan online environments shape communication with political outgroups. *Int. J. Commun.* **13**, 27 (2019).

12. R. K. Garrett, Echo chambers online?: Politically motivated selective exposure among Internet news users. *J. Comput. Mediat. Commun.* **14**, 265–285 (2009).

13. E. Bakshy, S. Messing, L. A. Adamic, Exposure to ideologically diverse news and opinion on Facebook. *Science* **348**, 1130–1132 (2015).

14. P. Barberá, J. T. Jost, J. Nagler, J. A. Tucker, R. Bonneau, Tweeting from left to right: Is online political communication more than an echo chamber? *Psychol. Sci.* **26**, 1531–1542 (2015).

15. E. Dubois, G. Blank, The echo chamber is overstated: The moderating effect of political interest and diverse media. *Inf. Commun. Soc.* **21**, 729–745 (2018).

16. G. Eady, J. Nagler, A. Guess, J. Zilinsky, J. A. Tucker, How many people live in political bubbles on social media? evidence from linked survey and twitter data. *SAGE Open* **9**, 215824401983270 (2019).

17. S. Flaxman, S. Goel, J. M. Rao, Filter bubbles, echo chambers, and online news consumption. *Public Opin. Q.* **80**, 298–320 (2016).

18. R. E. Robertson, S. Jiang, K. Joseph, L. Friedland, D. Lazer, C. Wilson, Auditing partisan audience bias within google search. *Proc. ACM Hum. Comput. Interact.* **2**, 1–22 (2018).

19. A. Guess, B. Nyhan, B. Lyons, J. Reifler, *Avoiding The Echo Chamber About Echo Chambers* (Mediawell, 2018).

20. A. M. Guess, (Almost) everything in moderation: New evidence on Americans' online media diets. *Am. J. Pol. Sci.* **65**, 1007–1022 (2021).

21. G. De Francisci Morales, C. Monti, M. Starnini, No echo in the chambers of political interactions on Reddit. *Sci. Rep.* **11**, 2818 (2021).

22. E. Peterson, S. Goel, S. Iyengar, Partisan selective exposure in online news consumption: Evidence from the 2016 presidential campaign. *Polit. Sci. Res. Methods* **9**, 242–258 (2021).

23. R. K. Garrett, The “echo chamber” distraction: Disinformation campaigns are the problem, not audience fragmentation. *J. Appl. Res. Mem. Cogn.* **6**, 370–376 (2018).

24. J. Yang, H. Rojas, M. Wojcieszak, T. Aalberg, S. Coen, J. Curran, K. Hayashi, S. Iyengar, P. K. Jones, G. Mazzoleni, S. Papathanassopoulos, J. W. Rhee, D. Rowe, S. Soroka, R. Tiffen, Why are “others” so polarized? Perceived political polarization and media use in 10 countries. *J. Comput. Mediat. Commun.* **21**, 349–367 (2016).

25. A. Bruns, Echo chamber? What echo chamber? Reviewing the evidence, in *6th Biennial Future of Journalism Conference (FOJ17)* (QUT, 2017).

26. A. Bruns, It’s not the technology, stupid: How the “Echo Chamber”and “Filter Bubble”metaphors have failed us, in *International Association for Media and Communication Research* (2019).

27. N. W. Robinson, C. Zeng, R. Lance Holbert, The Stubborn Pervasiveness of Television News in the Digital Age and the Field’s Attention to the Medium, 2010–2014. *J. Broadcast. Electron. Media* **62**, 287–301 (2018).

28. J. Allen, B. Howland, M. Mobius, D. Rothschild, D. J. Watts, Evaluating the fake news problem at the scale of the information ecosystem. *Sci. Adv.* **6**, eaay3539 (2020).

29. T. F. Baldwin, M. Barrett, B. Bates, Influence of cable on television news audiences. *Journal. Q.* **69**, 651–658 (1992).

30. J. G. Webster, Beneath the veneer of fragmentation: Television audience polarization in a multichannel world. *J. Commun.* **55**, 366–382 (2005).

31. J. T. Hamilton, The market and the media, in *Institutions of American Democracy: The Press*, G. Overholser, K. H. Jamieson, Eds. (Oxford Univ. Press, 2005), pp. 351–371.

32. E. F. Fowler, M. M. Franz, T. N. Ridout, The blue wave: Assessing Political advertising trends and democratic advantages in 2018. *Polit. Sci. Polit.* **53**, 57–63 (2020).

33. M. Prior, Media and political polarization. *Annu. Rev. Polit. Sci.* **16**, 101–127 (2013).

34. G. J. Martin, A. Yurukoglu, Bias in cable news: Persuasion and polarization. *Am. Econ. Rev.* **107**, 2565–2599 (2017).

35. M. P. McDonald, 2020 November General Election Turnout Rates (United States elections project, 2020); http://www.electproject.org/2020g [accessed 20 January 2022].

36. M. P. McDonald, Voter turnout demographics (United States Elections Project, 2020) [accessed 12 February 2022].

37. L. Molyneux, Multiplatform news consumption and its connections to civic engagement. *Journalism* **20**, 788–806 (2019).

38. A. Mitchell, J. Gottfried, M. Barthel, E. Shearer, *The Modern News Consumer* (Pew Research Center, 2016) [accessed 11 February 2022].

39. B. Oliphant, *10. Political engagement, knowledge and the midterms* (Pew Research Center–U.S. Politics & Policy, 2018).

40. K. E. Matsa, *Fewer Americans Rely On Tv News; What Type They Watch Varies By Who They Are* (Pew Research Center, 2018).

41. P. C. Meirick, Motivated misperception? Party, education, partisan news, and belief in "Death Panels". *Journal. Mass Commun. Q.* **90**, 39–57 (2013).

42. E. L. Kaplan, P. Meier, Nonparametric estimation from incomplete observations. *J. Am. Stat. Assoc.* **53**, 457–481 (1958).

43. E. J. Johnson, D. G. Goldstein, *Decisions By Default* (Princeton Univ. Press, 2013).

44. P. D. Meo, P. De Meo, E. Ferrara, G. Fiumara, A. Provetti, Generalized Louvain method for community detection in large networks, in the *11th International Conference on Intelligent Systems Design and Applications* (IEEE, 2011); https:/doi.org/10.1109/isda.2011.6121636.

45. G. J. Martin, J. McCrain, Local news and national politics. *Am. Polit. Sci. Rev.* **113**, 372–384 (2019).

46. H. Hosseinmardi, A. Ghasemian, A. Clauset, M. Mobius, D. M. Rothschild, D. J. Watts, Examining the consumption of radical content on YouTube. *Proc. Natl. Acad. Sci. U.S.A.* **118**, e2101967118 (2021).

47. R. Lewis, "This is what the news won't show you": YouTube creators and the reactionary politics of micro-celebrity. *Telev. New Media* **21**, 201–217 (2020).

48. J. Allen, M. Mobius, D. M. Rothschild, D. J. Watts, *Research Note: Examining Potential Bias in Large-Scale Censored Data* [Harvard Kennedy School (HKS) Misinformation Review, 2021]; https:/doi.org/10.37016/mr-2020-74.

49. R. Fletcher, C. T. Robertson, R. K. Nielsen, How many people live in politically partisan online news echo chambers in different countries? *J. Quant. Descr. Digit. Media* **1**, (2021).

50. D. B. Hindman, K. Wiegand, The big three's prime-time decline: A technological and social context. *J. Broadcast. Electron. Media* **52**, 119–135 (2008).

51. D. D'Alessio, M. Allen, Media bias in presidential elections: A meta-analysis. *J. Commun.* **50**, 133–156 (2000).

52. G. Smith, Sympathy for the Devil. *Am. Politics Res.* **45**, 63–84 (2017).

53. T. Groseclose, J. Milyo, A measure of media bias. *Q. J. Econ.* **120**, 1191–1237 (2005).

54. E. T. Gratz, M. E. Sarkees, M. P. Fitzgerald, Whose view is it anyway? Media coverage of litigation in for-profit firms' role in the opioid crisis. *J. Mark. Theory Pract.* 1–17 (2021).

55. Z. Xiao, W. Song, H. Xu, Z. Ren, Y. Sun, TIMME: Twitter ideology-detection via multi-task multi-relational embedding, in *Proceedings of the 26th ACM SIGKDD International Conference on Knowledge Discovery & Data Mining* (Association for Computing Machinery, 2020), pp. 2258–2268.

56. U.S. Census Bureau, *Educational Attainment of the Population 18 Years and Over, by Age, Sex, Race, and Hispanic Origin: 2019* (U.S. Census Bureau, 2020).

57. C. Davidson-Pilon, *Lifetimes* (2020); https://github.com/CamDavidsonPilon/lifetimes.
